# Enhancing lentiviral production for WAS gene therapy: a comparative analysis of stable producer cell lines evaluating flatware system and adherent bioreactors in perfusion mode

**DOI:** 10.3389/fbioe.2025.1648028

**Published:** 2025-09-05

**Authors:** Parameswari Singh, Nikki Indresh Lal, Monica Terrao, Sarah Schwingal, Martina Biserni, Florian Aeschimann, Andrea Strauch, Herbert Dersch, Angel Jaramillo, Andreas Gille, Holger Laux

**Affiliations:** ^1^ CSL Innovation GmbH, Marburg, Germany; ^2^ CSL Behring, Research, Bern, Switzerland; ^3^ Swiss Institute for Translational Medicine, Sitem-insel, Bern, Switzerland; ^4^ CSL Behring, Pasadena, CA, United States

**Keywords:** *ex-vivo* gene therapy, stable packaging cell line (PCL), lentivirus (LV), stable producer cell line, adherent bioreactors, perfusion process, hematopoietic stem cells

## Abstract

*Ex-vivo* gene therapies require scalable, high-quality lentivirus (LV) with excellent transduction efficiency. Achieving this involves a synergistic approach combining efficient vector design and LV process optimization. In our study, we evaluated transfection reagents for generating stable producer cell lines from two Tet-off regulated adherent stable LV packaging PCLs, GPRG and GPRTG, to produce lentivirus (LV) to treat Wiskott Aldrich Syndrome (WAS). Stable producer cell lines expressing the WAS transgene or GFP transgene were generated from GPRG and GPRTG PCLs. The GPRTG producer cell line showed 6-fold higher LV titer and resulted in better transduction of CD34^+^ cells. Further, we optimized the LV production process in continuous perfusion and recirculation mode and compared three technologies: traditional flatware systems, iCELLis™ Nano and scale-X™ Hydro Univercells adherent bioreactors using GPRTG stable producer cell line. Scale-X™ Hydro outperformed iCELLis™ Nano in LV productivity per surface area (TU/cm^2^). We successfully scaled up LV production from Scale-X™ Hydro (2.4 m^2^) to Scale-X™ Carbo (10 m^2^), producing 1.13E+12 TU per 10 m^2^ through 7 harvests using the continuous perfusion process. This process produced LV that efficiently transduced CD34^+^ cells, achieving a vector copy number (VCN) of upto 4 at a Multiplicity of Infection (MOI) of 10. Our study has successfully established a scalable, cost-effective and robust platform for LV production, demonstrating its potential for clinical applications.

## Introduction

Wiskott-Aldrich syndrome (WAS) is a severe X-linked immunodeficiency disorder caused by mutations in the WAS gene, characterized by thrombocytopenia, eczema, and increased risk of lymphoma and autoimmunity (Braun et al., 2013; Ochs et al., 2009). Hematopoietic stem cells (HSCs) are attractive targets for gene therapy for WAS and other hematological diseases, due to their inherent potential of genetic modification *ex-vivo*, and once transplanted, they support lifelong hematopoiesis in recipients (Logan et al., 2002; Tajer et al., 2024). Lentiviral vector (LV) based gene therapy leverages the delivery of LV-encoded genes into cellular genomic DNA to correct genetic disorders (Milone and O’Doherty, 2018). Human embryonic kidney (HEK293T) cells are extensively utilized for LV production (Broussau et al., 2023; Perry and Rayat, 2021). Transient LV production requires multiple plasmid transfection of HEK293(T) cells by chemical-based transfection methods that are highly sensitive to pH, imposes cell cytotoxicity, and presents significant challenges for scaling-up leading to batch variability and elevated costs (Coroadinha, 2022; Mccarron et al., 2017; Merten et al., 2016; Olgun et al., 2019; Shen et al., 2022). This emphasizes the need for practical, robust, and scalable viral vector production technologies that yield high-quality and cost-effective LV (Martínez-Molina et al., 2020). Stable LV production can be achieved with stable producer cell lines derived from packaging cell line (PCL) which express all necessary packaging elements for LV production and offer a more scalable and cost-effective alternative to transient transfection (Arrasate et al., 2024; Manceur et al., 2017; Sanber et al., 2015). Well-characterized examples are the Tet-inducible GPRG and GPRTG PCLs (Bonner et al., 2015). These PCLs are based on adherent HEK293T cells, modified by successive gamma retroviral transductions to introduce the viral genetic elements gag-pol, rev, tTA and VSV-G for GPRG cell line. The GPRTG PCL expresses the same viral genes as the GPRG PCL, as well as an additional viral gene tat (Bonner et al., 2015). Non-clonal producer cells (polyclonal pools) can be derived from PCLs by transfection of DNA encoding for the gene of interest (GOI). Achieving high transfection efficiencies is critical for reduced selection time and increased chance of high titer. Subsequently, monoclonal producer cell lines can be generated by isolating and propagating single cells from polyclonal stable pools. This study focused on optimizing cell line development comparing various transfection reagents for the generation of stable producer pools expressing GFP as a Gene of Interest (GOI). After identifying and selecting the most effective transfection reagent, the study compares GPRG-based producer cells with GPRTG-based producer cells expressing either the WAS gene or the GFP gene as the GOI, respectively. Adherent systems such as T flasks, multilayered CellSTACK and microcarriers have evolved to support LV production, however, they face limitations in scalability and reproducibility, along with an increased risk of contamination (Ausubel et al., 2012; Martínez-Molina et al., 2020; Van der Loo and Wright, 2016). Adherent bioreactors using stable producer cell lines enable scalable, robust and high-quality large-scale LV production. Fixed-bed bioreactor like iCELLis™ by Pall Corporation, offer a controlled, closed system for cell culture and virus production, maximizing cell growth and enhancing scalability of adherent cells (Knowles et al., 2013; Lennaertz et al., 2013). Similarly, Scale-X™ bioreactor system from Univercells Technologies™, is another fixed bed bioreactor that until now has been less explored for stable LV production (Berrie et al., 2020; Leinonen et al., 2020). Given that GFP (gene size: 720 bp) is a substantially smaller gene than WAS (gene size: 1,512 bp), the data obtained from GFP as the GOI are less representative when compared to WAS or other clinical candidates with larger gene sizes. Thus, we used the generated polyclonal and monoclonal stable GPRTG cell line expressing the WAS gene for adherent lentiviral (LV) process optimization studies, opting for the clinically desired gene from the start due to poor translatability of surrogates. This study compared lentiviral (LV) manufacturing performance in traditional flatware/cell stacks (CS) versus two adherent bioreactor systems (iCELLis™ and Univercells Technologies™). Process comparison assessed LV yield, scalability, metabolites and CD34^+^ transduction capacity. Additionally, CellSTACK one layer (CS1) flatware was evaluated for scalability to CellSTACK ten layer (CS10), iCELLis™ Nano (4 m^2^) was used for process optimization, and Scale-X™ Hydro (2.4 m^2^) was evaluated for its scalability to Scale-X™ Carbo (10 m^2^), further testing this scale for process robustness incorporating both polyclonal and monoclonal stable WAS producer cell lines and its subsequent applicability to GMP (Good Manufacturing Practice) LV production.

## Materials and methods

### Gene, reporter, antibiotic expression plasmids and parental stable packaging cells

The WAS transgene expression plasmid (pTL20c_MND_WAS_650) is a 3rd generation self-inactivating (SIN) LV expression plasmid. LV production of this HIV-1 based SIN LV expression plasmid (derived from the pCl20c plasmid backbone (Throm et al., 2009) is tetracycline-regulated with a mini cytomegalovirus (CMV) promoter and 7 tet operators (7tetO). The LV uses a MND (MPSV LTR, NCR deleted, dl587 PBS) promoter for WAS protein expression. The WAS cDNA was modified with two silent point mutations, C417A and C1098T, to remove SfiI restriction sites without altering the encoded amino acids. Downstream of the WAS cDNA is a Woodchuck Hepatitis Virus (WHV) Post-transcriptional Regulatory Element (WPRE) containing six point mutations to abrogate woodchuck hepatitis virus X (WHX) truncated protein expression while maintaining transgene expression (Zanta-Boussif et al., 2009). For WAS construct-1 (plasmid size: 9.6 kb, Supplementary Figure S1A), a 650 bp segment of the chicken hypersensitivity site 4 (cHS4) β-globin chromatin insulator (Urbinati et al., 2008) is inserted in the 3′LTR in reverse orientation to the viral transcript, serving as an additional safety and anti-silencing element. Variants of this LV construct were utilized in this study and differ in the sequence of the cHS4 insulator: WAS construct-2 (plasmid size: 9.6 kb, Supplementary Figure S1B) contains a mutated version of the 650 bp segment, WAS construct-3 (plasmid size: 9.3 kb, Supplementary Figure S1C) contains a 400 bp segment (Aker et al., 2007). An additional Enhanced Green Fluorescent Protein (EGFP) reporter transgene expression plasmid (pTL20c_MND_GFP_400, plasmid size: 8.3 kb) was utilized that differs in the encoded transgenic sequence, where EGFP replaces WAS. The antibiotic-resistance plasmid (pPGK_ble, plasmid size: 3.8kb, Supplementary Figure S1D) contains a Zeocin selection marker driven by PGK promoter.

### Concatemer generation from expression and selection marker plasmids

Plasmids (pTL20c_MND_WAS_650 or its variants, pTL20c_MND_GFP_400, pPGK_ble) were transformed into NEB^®^ Stable Competent *E. coli* Cells (a derivative of the DH10B strain) in LB Broth, Vegitone. Concatemers were generated with the respective transgene plasmid (pTL20c_WAS/ pTL20c_GFP) and the bleomycin resistance plasmid (pPGK_ble). SfiI and PfIMI restriction sites on pTL20c and pPGK_ble respectively, were used to linearize plasmids, which were then purified using Qiaquick gel extraction. The linearized plasmids of transgene plasmid and antibiotic resistance plasmid were directionally ligated in a molar ratio of 25:1. The concatemer mixture was purified using a Qiagen Genomic-tip 500/G column, eluted, precipitated in isopropanol, air-dried for 60 min, and resuspended in TAE buffer, to reach a usable concentration for experiments.

### Optimization of transfection conditions with GFP concatemer for stable cell line development

Three transfection reagents and varying amounts of concatemer were tested to identify the optimal condition for stable producer cell line development (CLD). The experiment first focused on selecting the best transfection reagent by comparing ViaFect™, Lipofectamine^®^ 3,000 and calcium phosphate by using a constant DNA concentration of 5 µg. GPRG PCL was seeded in 6-well plates at 1.33E+05 cells/cm^2^, 24 h before transfection. The growth medium used was Dulbecco’s modified Eagle’s medium with high glucose and Glutamax (DMEM, Gibco) with 10% Fetal Bovine Serum (FBS, USA origin Premium Corning, Gamma Irradiated), referred to as D10. The DMEM was supplemented with 1 ng/mL DOX (Doxycycline hyclate, MP Biomedicals; prepared in distilled water, Fisher Scientific) and is referred as D10+DOX. Transfection mixtures containing the GFP concatemer (5 µg per well) along with a transfection reagent (Lipofectamine™ 3,000 (kit with p3000™ enhancer reagent), ViaFect™ or CalPhos™) were preprared in OptiMEM without DOX. The transfection mixtures were added to cells and incubated for 4 h at 37 °C in OptiMEM (Reduced-Serum Media, Thermofisher) with 1 ng/mL DOX. Post-incubation, transfection media was replaced with D10+DOX. Transfection efficiency was determined by measuring % positive GFP cells and median fluorescence intensity (MFI) using a flow cytometer (MACSQuant Analyzer 16) at 72 h and 2 weeks post-transfection. Non-transfected cells were maintained as a negative control, to monitor the viability decline on host cell line during the selection phase. Zeocin (Zeocin selection antibiotic, Gibco; at 50 μg/mL) was introduced in cell culture media starting 72 h post-transfection for selection of stably transfected cells. Later different concatemer amounts such as 5 μg, 7.5 µg and 10 µg per well were tested with Lipofectamine 3,000™ transfection reagent. Transfection efficiency and median GFP fluorescence were examined 96 h and 1-month post-transfection to ensure stability of inserted DNA copies.

### LV material comparison between GPRG and GPRTG-based producer cell lines

GPRG and GPRTG PCLs were transfected in quadruplicates with WAS construct-1 concatemer and subjected to Zeocin selection for 21 days, resulting in stable polyclonal producer cells (pools) (four GPRTG-WAS pools and GPRG-WAS pools each). Similarly the two PCLs were transfected with the GFP concatemer, resulting in one GPRTG-GFP pool and GPRG-GFP pool each. These stable pools were seeded at a cell density of 2E+04 cells/cm^2^ in a single-layer CellSTACK (CS1) (Corning) with 100 mL of D10+DOX (growth phase). At 96 h post-seeding (hps), LV production was induced with a rinsing step of the cell layer with D10 to remove doxycycline, followed by addition of fresh D10. At 24 h post-induction (hpi), the supernatant was discarded and replaced with fresh D10, and the 1st harvest was collected 48 hpi, after which harvests were collected once every 24 h. Depending on extent of cell layer detachment, 6 to 10 LV harvests could be performed. In case of high cell detachment, harvests were centrifuged at 1,200 rpm for 3 min to have a cell-free harvest. Harvest samples were aliquoted and stored at −80 °C for subsequent analytical assays, including infectious titer (TU/mL), physical viral particle titer by p24 (vp/mL), RNA content (RNA copies/mL), RNA truncation analysis to check integrity and length of RNA sequences by ddPCR and VCN from CD34^+^ transduction studies.

### Generation of WAS polyclonal and monoclonal stable producer cell line, target locus amplication (TLA) analyses and stability assessment

The WAS construct-3 underwent comprehensive monoclonal cell line development utilising GPRTG packaging cells and transfection via the Lipofectamine™ 3,000 protocol. Cells derived from the GPRTG Master Cell Bank (MCB) were expanded and transfected with the WAS construct-3_zeocin concatemer using Lipofectamine™ 3,000, under two DNA input conditions (8.17 µg and 12.2 µg per 60 mm dish), resulting in the generation of eight polyclonal pools (L-1.1, L-1.2, L-1.3, L-1.4, L-2.1, L-2.2, L-2.3, L-2.4). Zeocin selection (80 μg/mL) commenced 72 h post-transfection, for all transfected pools and one non-transfected control. Following selection, stable polyclonal pools were expanded and cryopreserved as a Research Cell Bank (RCB) in a cryoprotectant solution of 90% FBS and 10% DMSO. To assess LV productivity, these stable polyclonal pools were expanded and induced in CS1 stacks, producing six harvests per pool, analyzed by ddPCR for infectious titer (TU_[ddPCR]_) quantification.

All stable polyclonal pools were subjected to a single-cell sorting workflow to capture cellular heterogeneity for monoclonal cell line derivation. Sorting was performed using the Cytena F.SIGHT™ system into 96 well-plates containing InstiGRO^®^ HEK in D10 medium supplemented with DOX and Zeocin. Plates were imaged using the Solentim™ imager on days 0, 3, 7, 11 and 13 post-sorting to confirm monoclonality. Clones were scaled from 96 well plate to CS5 (Cell Stack 5-layer) for monoclonal RCB cryopreservation. For LV productivity screening, 51 monoclonal clones were seeded into 6-well plates and the top four clones were selected based on monoclonality verification and TLA analyses of the RCB. The long-term stability of the four LV producer monoclonal cell clones was assessed over a cultivation period of 60–67 days. During this timeframe, each clone underwent 8 to 9 independent LV production runs (P1 to P8/P9), with three sequential harvests per production, to evaluate consistency in infectious titer output. TLA sequencing was performed on the top performing clone to confirm vector integrity, identify integration sites, detect sequence variants and estimate the integrated vector copy number. Non-induced cells from the seed train of the stability study were analyzed by TLA sequencing at time point 0 (from RCB vials, referred to as TLA day0), and day 43 (TLA day43). LV genome integrity and variant detection were further assessed by Next-Generation Sequencing (NGS) using harvest 2 from LV production 1 (P1) and production 6 (P6) during the stability study. The RNA was reverse transcribed into cDNA and sequenced by NGS to evaluate consistency across the production timeline.

### LV production and optimization in flatware (CS1) and pilot scale (CS10)

One GPRTG-WAS pool was used for process optimizations and technological evaluations. LV production optimization was conducted in CS1, with the best conditions validated in 10-layer CellSTACK (CS10). Variables such as seeding densities, induction with or without a wash step, surface coating, and harvest media volume (0.24 mL/cm^2^ vs. 0.16 mL/cm^2^) were examined. For coating experiments, CS1 was coated with Poly-L-lysine solution of high and low molecular weight (P4832 Sigma Aldrich and A-005-M Merck). For a seeding density of 8.5E+04 cells/cm^2^, induction was performed 24 h post-seeding (hps). Lower seeding densities (1.3E+04 and 2E+04 cells/cm^2^) required 72–96 hps before induction.

To examine upscaling differences, four conditions (1.3E+04 and 2E+04 cells/cm^2^ with and without wash at induction) were performed in both CS1 and CS10 for yield comparison. The first at-scale LV production in 16 CS10 units was performed at a seeding density of 8.5E+04 cells/cm^2^, with LV production induced at 24 hps. Following CS1 optimization, the process was applied to another 16 CS10 batch for comparison where CS10 were seeded at 2E+04 cells/cm^2^, with LV production induced at 96 hps without wash. Harvest was collected every 24 h, starting at 48 h post-induction, and stored at −80 °C for analysis.

### Perfusion process optimization for LV production in small-scale fixed-bed bioreactors

#### iCELLis™ nano bioreactor (4 m^2^)

iCELLis™ Nano 4 m^2^ (Pall BioTech), is a high compaction (144 g/L) fixed-bed bioreactor with a 10 cm bed height, 0.2 L volume, and final working volume of 800–850 mL. It features probes for temperature, pH, dissolved oxygen (DO), and biomass monitoring. The vessel was filled with pre-warmed D10 media and equilibrated for 4 h before inoculation. Cells were inoculated at 2E+04 cells/cm^2^ in a controlled environment with key parameters such as temperature at 37 °C, stirrer speed of 1,170 rpm, DO set point of 50% with 30 mL/min airflow and pH set point of 7.4 or 7.2, controlled by CO_2_ gassing and 0.5 N NaOH addition.

LV production was tested in six iCELLis™ runs (iC01-iC06) using the same producer cell line for comparability with process parameters summarized in Table 1. The LV process included three phases: inoculation and growth (96 h post-inoculation), induction for LV production, and LV harvest collection. Three methods were evaluated: 1. Continuous perfusion without vessel wash at induction (iC01-iC03) with pH set points 7.2/7.4 and 0.16 mL/cm^2^/day perfusion volume, 2. Recirculation (iC04) with 0.19 mL/cm^2^/day media, 3. Perfusion with vessel volume exchange (iC05-iC06).

**TABLE 1:** Comparison of process type and its pH set point, base/ vessel wash.

Run	Process type	Media volume	pH set point	Base	Vessel wash
iC01	Perfusion	Std, 0.16 mL/cm^2^	7.2	yes	no
iC02	Perfusion	Std, 0.16 mL/cm^2^	7.2	no	no
iC03	Perfusion	Std, 0.16 mL/cm^2^	7.4	no	no
iC04	Recirculation	High, 0.19 mL/cm^2^	7.2	no	no
iC05	Perfusion	Std, 0.16 mL/cm^2^	7.2	no	yes
iC06	Perfusion	Std, 0.16 mL/cm^2^	7.2	no	yes
S05	Recirculation	High, 0.19 mL/cm^2^	7.4	no	no
S08	Perfusion	High, 0.28 mL/cm^2^	7.2	no	yes

LV induction typically began on day 4 when cells reached >1.80E+05 cells/cm^2^. The first perfusate was discarded 24 h post-induction (hpi). Harvests were collected continuously at 0.16–0.19 mL/cm^2^/day, starting from 48 hpi, with media supply maintained and harvest bags changed every 24 h. In recirculation mode, media lines were connected to a recirculation bottle, and harvests were collected by emptying and refilling the bottle. Harvest aliquots were frozen at −80 °C for analysis.

#### Scale-X™ hydro bioreactor (2.4 m^2^)

The Scale-X™ Hydro 2.4 m^2^ bioreactor, with an 800 mL working volume and 2.4 m^2^ surface area for cell attachment, features a fixed bed of alternating layers of non-woven PET fabric and polyvinyl spacer netting. Media flow is driven by a magnetic stirrer. Process parameters such as pH, temperature, and target induction density mirrored those of the iCELLis™ Nano, with a seeding density of 1.3E+04 cells/cm^2^ and stirrer speed adjusted to 850–900 rpm.

LV production was tested in two runs (S05, S08). S05 operated in recirculation mode without vessel volume exchange, while S08 used perfusion mode with vessel volume exchange at induction. In recirculation mode, media was recirculated four times in 24 h during the 4-day growth phase with induction on day 4 (cell density >1.80E+05 cells/cm^2^). Harvests were collected continuously at 0.19 mL/cm^2^/day for S05 and 0.28 mL/cm^2^/day for S08, with media supply maintained and harvest bags changed every 24 h. Aliquots were frozen at −80 °C for analysis.

### Scale-up and pilot-scale production of LV in fixed-bed bioreactor (Scale-X™ carbo 10 m^2^)

The Scale-X™ Carbo 10 m^2^ (R&D grade) versions V1, V1.4, and V2 were utilized for pilot-scale LV production. This fully automated system is equipped with pH, DO, and temperature probes. Initial runs (SC01, SC02) used WAS construct-1 producer cells, followed by runs with WAS construct-2 and 3 to assess robustness.

The R&D grade allows for fixed-bed carrier sampling of 11.5 cm^2^ each, and cell count by cell lysis. V1 and V1.4 were single-use vessels assembled with calibrated pH and DO probes for autoclaving, while vessels of V2 were gamma-irradiated ready-to-use with single-use pH and DO probes. All vessels were filled with 1.6 L of media post-sterilization The pH was set to 7.2 ± 0.05, temperature to 37 °C, and stirrer speed to achieve 1.1–1.3 cm/s linear speed. DO was maintained at 50% with 200 mL/min air or O_2_ supply.

After media equilibration and pH confirmation (7.2–7.3), the bioreactor was inoculated at 1.3E+04 to 2.2E+04 cells/cm^2^ with a final volume of 1.8 L. Recirculation of 19 L of media at 40 mL/min was initiated for 4-day growth phase after confirmation of >95% of cell attachment (4–24 hps), to ensure adequate nutrient supply for cells. Induction for LV production was performed, without wash, at 96 hps or upon reaching >1.80E+05 cells/cm^2^. The perfusion process included continuous harvest collection at 4 °C and constant media feed, similar to small-scale perfusion process. Target perfusion volume during induction was 23L (26L for V2), 19L (for all versions) at Harvest 1 and 23L (26L for V2) for subsequent harvests. Media and harvest bags were changed every 24 h, with LV aliquots frozen at −80 °C for analysis.

### Infectious titer determination by flow cytometry

HEK293T/17 cells were cultured in DMEM medium supplemented with 10% FBS and 1% Penicillin-Streptomycin (Thermo Fisher). Prior to transduction, cells were seeded at a density of 1E+05 cells/well into 24-well plates using DMEM medium supplemented with 12.5 μg/mL polybrene (Merck). LV supernatants were serially diluted and 200 µL of each dilution was added to seeded cells. Four days post-transduction, cells were trypsinized, stained with LIVE/ DEAD Fixable Aqua Dead Cell Stain Kit (Thermo Fisher Scientific), fixed and permeabilized with BD Cytofix/Cytoperm™ Fixation/Permeablization Kit (BD Biosciences). The WAS protein (WASp) staining was performed by incubating cells with mouse anti-WAS 5A5 antibody (BD Biosciences) at 1:100 dilution and goat anti-mouse APC conjugated (Thermo Fisher Scientific) at dilution of 1:1,000. The WASp-positive cells were determined using MACSQuant^®^ 16 Flow Cytometer (Miltenyi Biotec) and infectious titers were calculated in TU/mL.

### Physical titer and truncation determination by digital droplet polymerase chain reaction (ddPCR)

Samples were treated with DNase (RNAse free DNAse from Qiagen™) to remove host cell genomic DNA contamination. Viral RNA was purified using the QIAamp Viral RNA Mini Kit and reverse-transcribed into cDNA using the High-Capacity cDNA Reverse Transcription Kit (Thermo Fisher Scientific). Samples were analysed by ddPCR using specific primers and probes according to the lentivirus genome region to be analyzed. The physical titer is reported in copies/mL which allows the deduction of the number of lentiviruses as each virus contains two RNA copies. To understand potential differences between packaging cell lines based on incomplete or truncated sequences, RNA truncation was evaluated and here results are reported as a ratio between the 3′ end (insulator region) and the 5′ end (U5 psi region).

### Physical titer determination by p24 ELISA

The p24 ELISA was performed using the QuickTiter™ LV Titer Kit (Cell Biolabs) that quantifies only the LV-associated p24. Samples were subjected to a pull-down step to collect LV particles, followed by serial dilution and sandwich ELISA with FITC-conjugated anti-p24 and HRP-conjugated anti-FITC antibodies at 1:1,000 dilution. Results were analyzed using a Tecan Spark reader and reported as ng/mL of p24, with physical viral particle titers calculated with the formula from the kit manual [1 ng/mL p24 = 1.25E+07 LV particles (vp/mL)].

### Concentration of LV from harvests and human CD34^+^ cells transduction

Two harvests each from GPRG-WAS pools and GPRTG-WAS pools from small scale CS1 were processed individually: GPRG-WAS Pool 1 (harvests 3 and 4), GPRG-WAS Pool 2 (harvests 3 and 4), GPRG-WAS Pool 3 (harvests 3 and 4), GPRG-WAS Pool 4 (harvests 3 and 4), GPRTG-WAS Pool 1 (harvests 3 and 4), GPRTG-WAS Pool 2 (harvests 2 and 3), GPRTG-WAS Pool 3 (harvests 3 and 4), GPRTG-WAS Pool 4 (harvests 2 and 3). Harvest 2 and 3 were taken for GPRTG-WAS Pool 2 and 3 due to noticeable cell detachment in CS1. Harvest material from CS1s was purified by 20% sucrose density gradient ultracentrifugation at 25,000 rpm for 2 h at 4 °C. The viral pellet was resuspended in X-VIVO10 (Lonza) supplemented with 2% human serum albumin (CSL Behring), and incubated at 4 °C for 2 h. Cryopreserved G-CSF Mobilized Human Peripheral Blood CD34^+^ Cells (Cat# M34C-GCSF-2, Hemacare Bioresearch Products, US) from a male donor were thawed and cultured in a prestimulation media containing serum-free X-VIVO10 medium (Cat# LZ-BE04-743Q, Lonza, Basel, Switzerland), 1X GlutaMax CTS (Cat# A12860-01, Gibco, Thermo Fisher Scientific, US), 2% Human Serum Albumin (CSL Behring, Switzerland), 100 ng/mL rhTPO (Cat# 1017–050), 300 ng/mL rhSCF (Cat# 1018–050), 200 ng/mL rhFlt-3 (Cat# 1015–050) (Cell Genix Technologies, Germany) for 24 h at 37 °C and 5% CO_2_ at 2E+06 cells/mL. After 24 h, cells were seeded at 2E+05 cells/0.1 mL in a 96-well plate, and the prestimulation media was changed to a fresh medium containing the same cytokines and the following Transduction Enhancers (TEs): 1 mg/mL LentiBOOST (Cat#SB-A-LF-901–01, SIRION Biotech, Germany), 10 μM PGE2 (Cat# 72372, Stem Cell Technologies, Canada), 8 μg/mL Protamine Sulfate (Cat# P3369-10G, Sigma Aldrich, US). Following 1 h pre-incubation with TEs, CD34^+^ cells were transduced with LV at a MOI of 1, 5 and 10 and returned to the incubator for 24 h. Post-transduction, cells were washed and resuspended in expansion media (X-VIVO10, GlutaMax, 2% human serum albumin, rhTPO, rhSCF, rhFlt-3) for 24 h. Cells were then transferred to a 12-well plate with fresh expansion media. VCNs were evaluated by ddPCR at 8 days after transduction.

For pilot-scale comparison, LV from Scale-X™ Carbo 10 m^2^ (SC01) and 16 CS10 R2 were processed in Sublot 1 (combined harvests 1 and 2) and Sublot 2 (combined harvests 3 and 4). They were purified using Mustang Q anion exchange membrane and concentrated by tangential flow filtration (TFF) (Vogel et al., 2024) and VCN was analysed by ddPCR.

### Vector copy number quantification

Vector copy Number (VCN) was assessed by ddPCR using QX200™ Droplet Digital PCR (Bio-Rad Laboratories, Germany) according to manufacturer’s instructions. In brief, the reaction mixture (20 μL) containing 50 ng template DNA, 1X ddPCR Supermix for Probes [(No deoxyuridine triphosphate (dUTP) (Bio-Rad)], 50 units/μL of HaeIII restriction enzyme (Cat# R0108M, New England Biolabs, US) 900 nM of each primer, and 250 nM of each probe (Bio-Rad) was loaded into the sample well in the QX100 Droplet Generator (Bio-Rad). A total of 40 μL of oil–water emulsion containing approximately 20,000 droplets was generated with the droplet generator. Post-PCR amplification, droplets were analyzed using the QX200™ Droplet Reader and QuantaSoft software (Bio-Rad). The following primer-probe sets were used to amplify lentiviral psi (Ѱ) and the human ribonuclease P protein subunit p30 (RPP30) as a control for normalization (Ѱ: fwd 5′-TAG​TGT​GTG​CCC​GTC​TGT​TG-3′, rev 5′-CCT​CTG​GTT​TCC​CTT​TCG​CT-3′ and probe 5′-FAM-TCTCTAGCAGTGGCGCCCGA-3’; RPP30: fwd 5′-TGT​AAG​TGG​TAG​TGC​ATA​GAC​TTT​A-3′, rev 5′- GTC​AAG​AGT​AGG​AGG​ACA​TTT​GA-3′ and probe 5′-HEX- AGG​CAG​ACT​GAC​ACT​AGA​GTT​CAC-3′). VCNs were determined by calculating the number of copies of Ѱ to every two copies of RPP30.

## Results

### Lipofectamine™ 3000 with p3000 enhancer reagent achieves superior transfection efficiency in producer cell line generation

**FIGURE 1:**
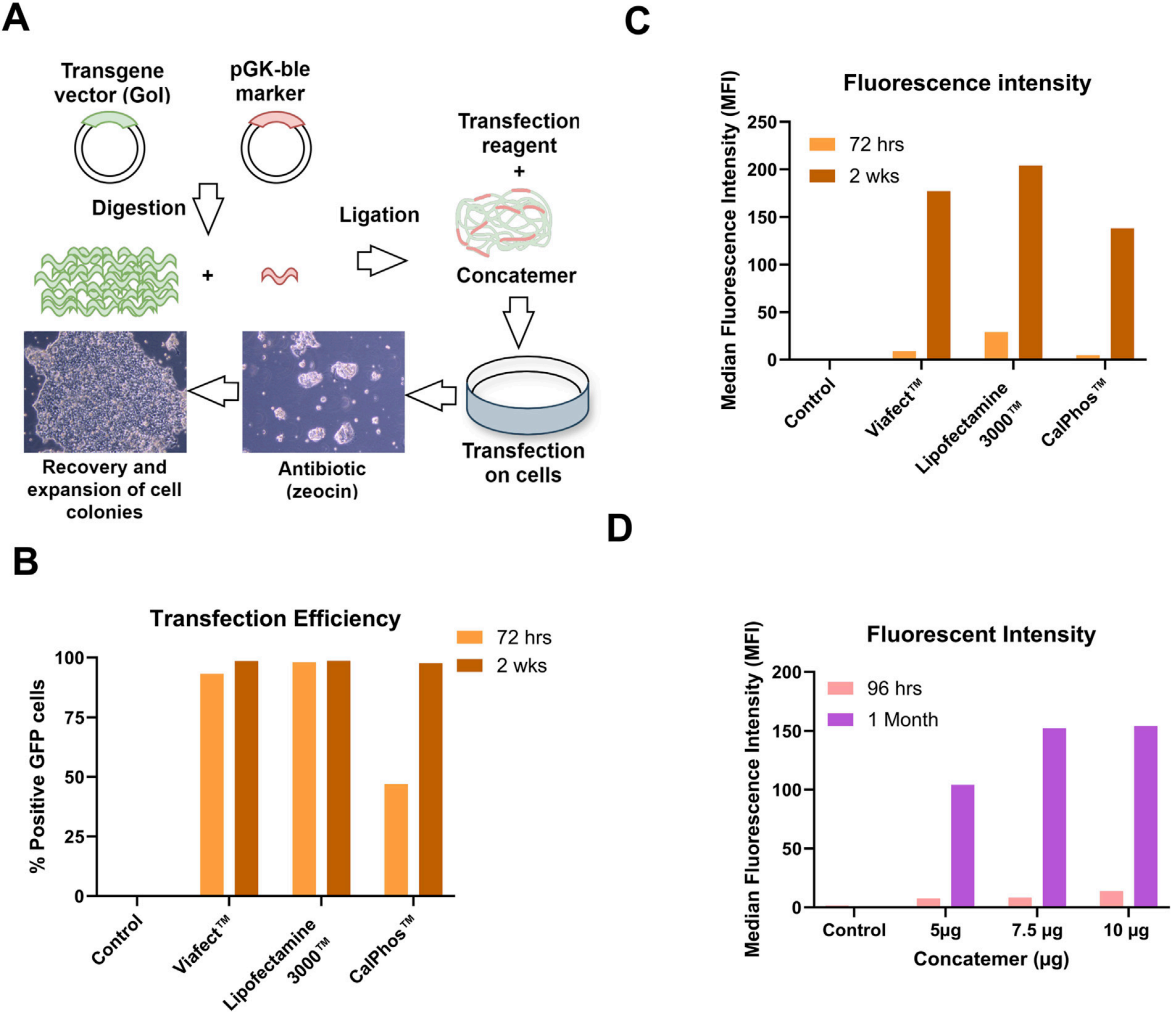
Optimization of Transfection Conditions with GFP Concatemer for Stable Cell Line Development. **(A)** Workflow of concatemeric array formation, transfection of PCL and antibiotic selection for generating stable producer cells. **(B)** Transfection efficiency measured as % Positive GFP cells. **(C)** Median Fluorescence intensity (MFI) measured at 72 h post-transfection and 2 weeks post-Zeocin selection. **(D)** GFP expression assessed as MFI using Lipofectamine™ 3,000 with p3000 enhancer at different concatemer amounts at 96 h and 1-month post-transfection and selection.

The graphical workflow for the generation of stable producer cell lines is illustrated in Figure 1A showing concatemer generation and transfection and selection of cells with Zeocin antibiotic. To determine the optimal transfection conditions for generating stable producer cell lines, GPRG PCLs were transfected with GFP concatemer (long continuous DNA containing 25 copies of GFP and one copy of Zeocin resistance gene) using ViaFect™, Lipofectamine™ 3,000 (with p3000 enhancer reagent), and CalPhos™. Flow cytometry analysis at 72 h post-transfection showed that cells transfected with ViaFect™ and Lipofectamine™ 3,000 had >95% GFP positive cells, while the CalPhos™ condition only achieved 50% (Figure 1B). Afterwards, cells were cultured with Zeocin for 2 wks to select for stable integration reaching a cell viability above 90% and all three transfection reagents resulted in a 100% GFP-positivity (Figure 1C). The median fluorescence intensity (MFI) increased following the 2-wks selection period, with the highest intensity observed in cells transfected with Lipofectamine™ 3,000 (Figure 1C). It was observed that selection period of 2 weeks were not sufficient (upon observing non-transfected control cells) and in upcoming experiment, cells were monitored upto 1 month in Zeocin selection.

Different concentrations of concatemer (5 μg, 7 μg, and 10 µg) were analyzed to determine the optimal concentration that enhances transfection efficiency and LV titer while ensuring it is not toxic to cells. The optimal amount of concatemer for Lipofectamine™ 3000-mediated transfection was determined to be between 7.5 µg and 10 µg. This range was identified based on the high fluorescence intensities observed at both 96 h and 1-month post-transfection and Zeocin selection (Figure 1D). To this end, cells were seeded and induced for lentiviral production. Elevated GFP LV titers were observed at higher concatemer DNA concentrations, reaching up to 4.45E+06 TU/mL in pooled LV harvests for 10 μg and 4.385E+06 TU/mL for 7.5 µg, compared to 1.105E+06 TU/mL for the 5 µg condition. Lipofectamine™ 3,000 was used in all upcoming experiments to generate stable producer cell lines with WAS constructs 1, 2 and 3.

### GPRTG-WAS pools demonstrate superior LV production and quality compared to GPRG-WAS pools

Prior to generating a stable producer cell line for *ex-vivo* gene therapy applications, it was imperative to select one suitable PCL, for producing good quality LV expressing the transgene. For this, stable producer pools namely GPRG-WAS pools (n = 4), GPRTG-WAS pools (n = 4), GPRG-GFP pool (n = 1) and GPRTG-GFP pool (n = 1) were generated by transfection with WAS construct-1 concatemer (long continuous DNA containing 25 copies of WAS and one copy of Zeocin resistance gene) or GFP concatemer (long continuous DNA containing 25 copies of GFP and one copy of Zeocin resistance gene) respectively. Cells were subsequently selected with Zeocin and seeded in CS1 for LV production. Overall, 10 harvests were achieved for GPRG-WAS pools and the GPRG-GFP pool, while 6 and 8 harvests were obtained for GPRTG-WAS pools and the GPRTG-GFP pool, respectively, due to cell detachment.

**FIGURE 2:**
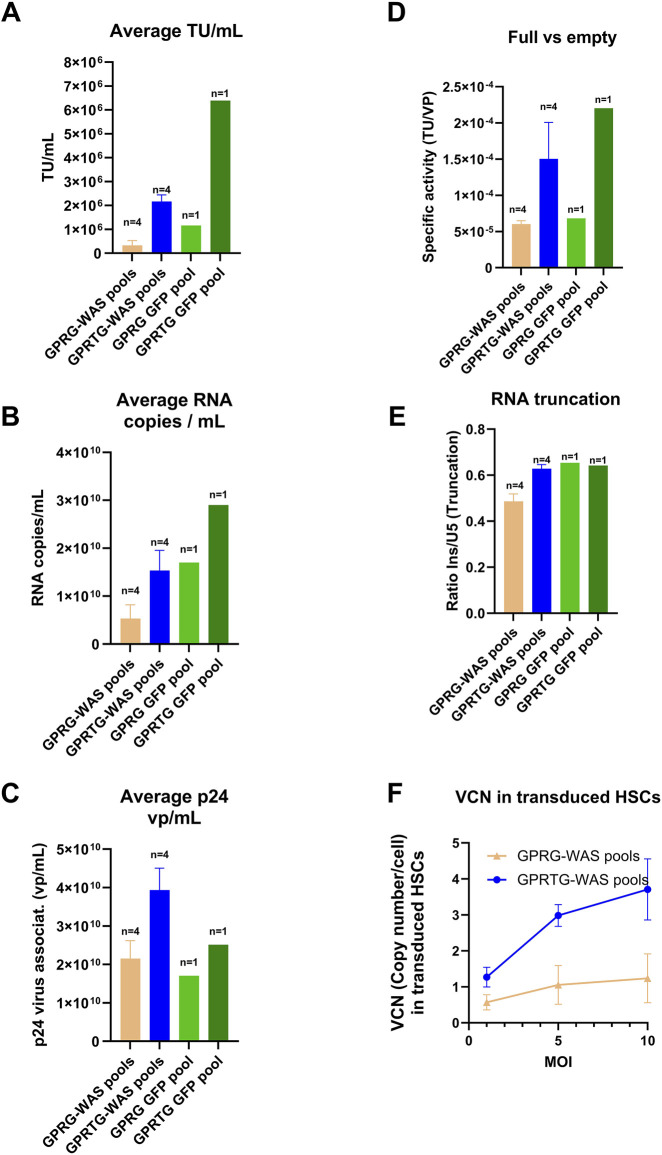
Comparison of LV generated from GPRG and GPRTG stable pools (WAS producer pools and GFP producer pools). Data represents an average of 4 pools for GPRG-WAS and GPRTG-WAS respectively along with standard deviation (SD). **(A)** Infectious LV titer. **(B)** Physical LV titer by RNA copies. **(C)** Physical LV titer by virus-associated p24. **(D)** Full vs. empty LV specific activity (TU/VP): analysed as ratio of infectious unit (TU)/ Physical LV titer by RNA copies/mL. **(E)** LV RNA truncation factor: Analyses conducted based on CS1 harvest availability influenced by cell detachments. Harvest 1, 4, 7, 10 were analysed from GPRG-WAS pools and GPRG-GFP pools, harvest 1 and 4 from GPRTG-WAS pools, harvest 1, 4, 7 for GPRTG GFP pools. **(F)** LV transduction capacity in HSCs. LV from GPRG-WAS and GPRTG-WAS stable pools was used to infect CD34^+^ HSCs at MOIs of 1, 5 and 10. VCN was determined as described in M&M. Shown are means +/- SD for GPRG-WAS pools (n = 4) and GPRTG-WAS pools (n = 4).

GPRTG-WAS pools yielded more infectious LV, with an average total TU of 1.25E+09 (calculated by total TU from all harvests per pool per CS1 production, then average of all 4 pools) compared to 3.25E+08 average total TU for GPRG-WAS pools. The average TU/mL per harvest (average of TU/mL across all harvest and 4 pools) was 6.6-fold higher for GPRTG-WAS pools (2.17E+06 Avg TU/mL, n = 4) compared to GPRG-WAS pools (3.26E+05 Avg TU/mL, n = 4). Similarly, a 5.5-fold difference was observed between GPRTG-GFP pools (1.16E+06 Avg TU/mL per harvest, n = 1) and GPRG-GFP pools (6.39E+05 Avg TU/mL per harvest, n = 1) (Figure 2A).

The infectious titer showed the same trend as the genomic titer. The genomic titer for the GPRTG-WAS pools measured by ddPCR was 3-fold higher (1.54E+10 RNA copies/mL per harvest, n = 4) compared to GPRG-WAS pools (5.28E+09 RNA copies/mL, n = 4). The same tendency was identified also for the GFP candidate with a 1.7-fold higher genomic titer for GPRTG-GFP in comparison to GPRG-GFP pools (n = 1) (Figure 2B).

The physical viral particle titer (vp/mL) derived from virus-associated p24 measurement is also having the same trend. It was 1.8-fold higher by GPRTG-WAS pools (3.9E+10 vp/mL, n = 4), compared to GPRG pools (2.15E+10 vp/mL, n = 4). A similar fold change was observed for the GFP candidate (1.7 fold change) (Figure 2C). The comparison of GOI-containing versus GOI-deficient LV particles showed a 3.6-fold increase in GPRTG-WAS pools compared to GPRG-WAS pools (n = 4), and a 4.6-fold increase in GPRTG-GFP pools compared to GPRG-GFP pools (n = 1) (Figure 2D). As shown in Figure 2E, the average RNA truncation [ratio between the 3′ end (insulator region) and the 5′ end (U5 psi region)] evaluated by ddPCR for both cell lines did not significantly change between GPRG and GPRTG pools (WAS and GFP constructs).

Transduction experiments on CD34^+^ cells at MOI of 1, 5, and 10 demonstrated a dose-dependent response for LV material produced by GPRTG-WAS pools (n = 4). A VCN of 1 was achieved at an MOI of 1, and subsequent higher VCNs of up to 3 and above were achieved at MOIs of 5 and 10. This dose-response trend was not observed with LV produced from GPRG-WAS pools (n = 4), where VCN of 1 was achieved at MOI of 5, and no further increase at higher MOIs (Figure 2F). These results highlight the superior performance of GPRTG-WAS pools compared to GPRG-WAS pools, emphasizing the importance of selecting the appropriate PCL for Cell Line Development (CLD) projects. Infectious titers were determined by transducing HEK293T cells with serial dilutions and analyzing transgene expression via flow cytometry. The MOI was calculated based on infectious titer (TU/mL) using the formula: MOI = [(volume of LV added in mL) × (infectious titer in TU/mL)]/ (number of cells seeded).

### Stable monoclonal WAS lentiviral producer clones demonstrate robust long-term productivity and genomic integrity

**FIGURE 3:**
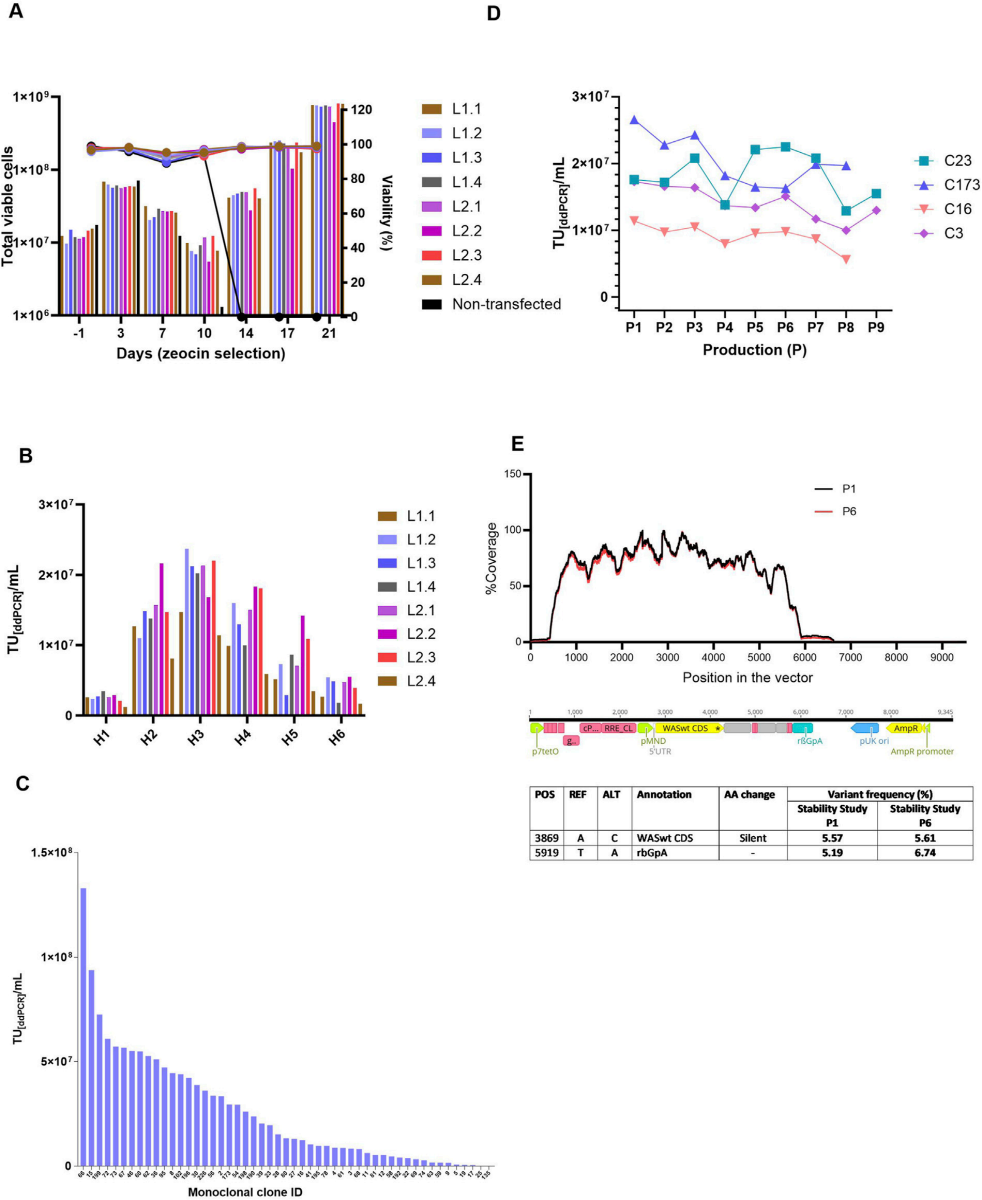
Polyclonal and Monoclonal cell line generation and clone characterization. **(A)** Total viable cells in culture along with viability (%) of polyclonal pool cells that were under zeocin section pressure. A drop in total cell numbers for all pools was seen on Day 10, coinciding with the death of all non-transfected cells (as shown by control), after which enhanced cell recovery was seen for stable pools. **(B)** Infectious titers, expressed in TU_[ddPCR]/_mL, were determined by ddPCR for individual lentiviral harvests obtained from polyclonal pools. Peak expression was seen in H3 reaching 2.37E+07 TU per mL for pool L2.2. H: Harvest, 1 to 6 indicates harvest days 48h post-induction. **(C)** Infectious titer (TU_[ddPCR]/_mL) of monoclonal cell clones ranked based on titer, with clone IDs displayed on the X-axis. The highest titer was 1.33E+08 TU/mL from clone 66. **(D)** Stability study on clones. Infectious titers from pooled harvests (H1 to H3) either done as technical replicates n = 2/n = 3 with respect to LV productions (P) throughout 8 -9 productions (P8/P9). **(E)** Shows the % coverage with respect to position of the vector sequence (bp) for harvest 2 of production 1 (P1) and production 6 (P6). Table presents the LV NGS sequencing analyses, detailing the vector fragment annotation and corresponding amino acid exchanges for both P1 (LV Production 1) and P6 (LV Production 6) from the stability study. POS: Position in the reference sequence (pBRNGTR319_pTL20c_MND_WAS_650_5xSA12345mut); REF: Reference nucleotide; ALT: Altered nucleotide; AA change: amino acid change/ impact). p7tetO: Tet-inducible promoter RRE: Rev response element, pMND: MND promoter, WASint CDS: WAS gene coding sequence, rBGpA: Rabbit β-globin polyA signal, pUK ori: Plasmid origin of replication, AmpR promoter: Ampicillin resistance promoter.

Monoclonal stable producer cell lines were derived from WAS-stable polyclonal pools via lipofectamine method using WAS-Zeocin concatemer. To establish zeocin-resistant polyclonal stable pools, both transfected and non-transfected control cells were subjected to continuous zeocin selection for 21 days. Complete cell death in non-transfected controls by day 21 validated the efficacy of antibiotic selection. Cell proliferation and viability were rigorously monitored throughout the selection period. A pronounced decline in viable cell density, termed the crisis point, was observed at day 10 post zeocin exposure, followed by a recovery in cell viability in all stable pools (Figure 3A). In contrast, non-transfected controls showed no recovery. Total viable cell counts and viability percentages are shown in Figure 3A. Infectious titers from the stable polyclonal pools were quantified via droplet digital PCR (ddPCR). As shown in Figure 3B, the titers of individual harvests are reported as TU_[ddPCR]/_mL for each pool. All pools except L1.1 and L2.4 exhibited high lentiviral titers, exceeding 2.0E+07 TU_[ddPCR]/_mL at harvest 3. Polyclonal pools were subjected to single-cell sorting to derive monoclonal cell lines. 51 monoclonal clones exhibiting robust proliferation were assessed for lentiviral vector productivity. The lentiviral titers among the clones were heterogeneous, as illustrated in the Figure 3C, with the highest titer observed in clone 66, reaching 1.33E+08 TU/mL.

To verify stability and integrity of the genomic integration, Targeted Locus Amplification (TLA) analysis was performed on the generated RCBs derived from the monoclonal clones. Clones 23 and 173, which lacked sequence variants within the vector genome, were prioritized for further analysis. Clones 16 and 3 were also included, as no mutations were identified in the WAS coding region or the insulator. Based on TLA results, lentiviral titers, and cell growth profiles, clones 23, 173, 16, and 3 were chosen for long-term stability assessment. Throughout a 60/67 days stability study, all five evaluated monoclonal producer cell lines exhibited comparable morphological characteristics, with no significant differences in confluency or detachment observed across LV production runs. Cell growth remained robust and consistent, with surface cell densities ranging from 4E+05 to 6E+05 cells/cm^2^ and viabilities exceeding 95% throughout the culture period. Infectious titer analysis shown in Figure 3D) revealed minor inter-clonal variability. Clone 173 maintained stable performance across eight production runs and yielded the highest mean titer at 2.1E+07 infectious particles across eight productions. In contrast, Clone 214 exhibited the lowest mean titer at 7.03E+06 infectious particles over nine productions. Three of the five clones demonstrated stable LVV production, with approximately ≤25% titer reduction over the study duration. The remaining two clones showed a decline of approximately 50% in infectious titer, indicating reduced production stability.

TLA samples (non-induced cells) were collected at Days 0 (RCB), 43, and 60 from non-induced cells, corresponding to theoretical timelines for MCB (43 days) and WCB (60 days) generation including Univercells 10 m^2^ manufacturing processes. TLA sequencing of clone 173 at Days 0 and 43 confirmed a single integration site on chromosome X with no sequence variants. In parallel, lentiviral vectors (LVs) harvested from productions P1 and P6 were subjected to next-generation sequencing (NGS) to evaluate sequence fidelity over time. The analysis revealed high sequence identity across the full-length LV genome, confirming genetic stability between early (P1) and late (P6) production stages. Figure 3E summarizes the NGS analyses, including the annotated vector regions and corresponding amino acid substitutions for variants with a frequency above 5%.

Two nucleotide variants were identified at positions 3,869 and 5,919 in both P1 and P6 samples, each with a frequency exceeding 5%. These variants were determined to be either silent or located outside the WAS coding region and, therefore, are not expected to affect the functionality or integrity of the LV product. Overall, the results indicate consistent genetic stability of the LV genome during the extended culture period, supporting the robustness of the selected monoclonal clones for long-term lentiviral vector production.

### Optimization of seeding density and induction conditions enhances LV production efficiency and reproducibility in CellSTACK

**FIGURE 4:**
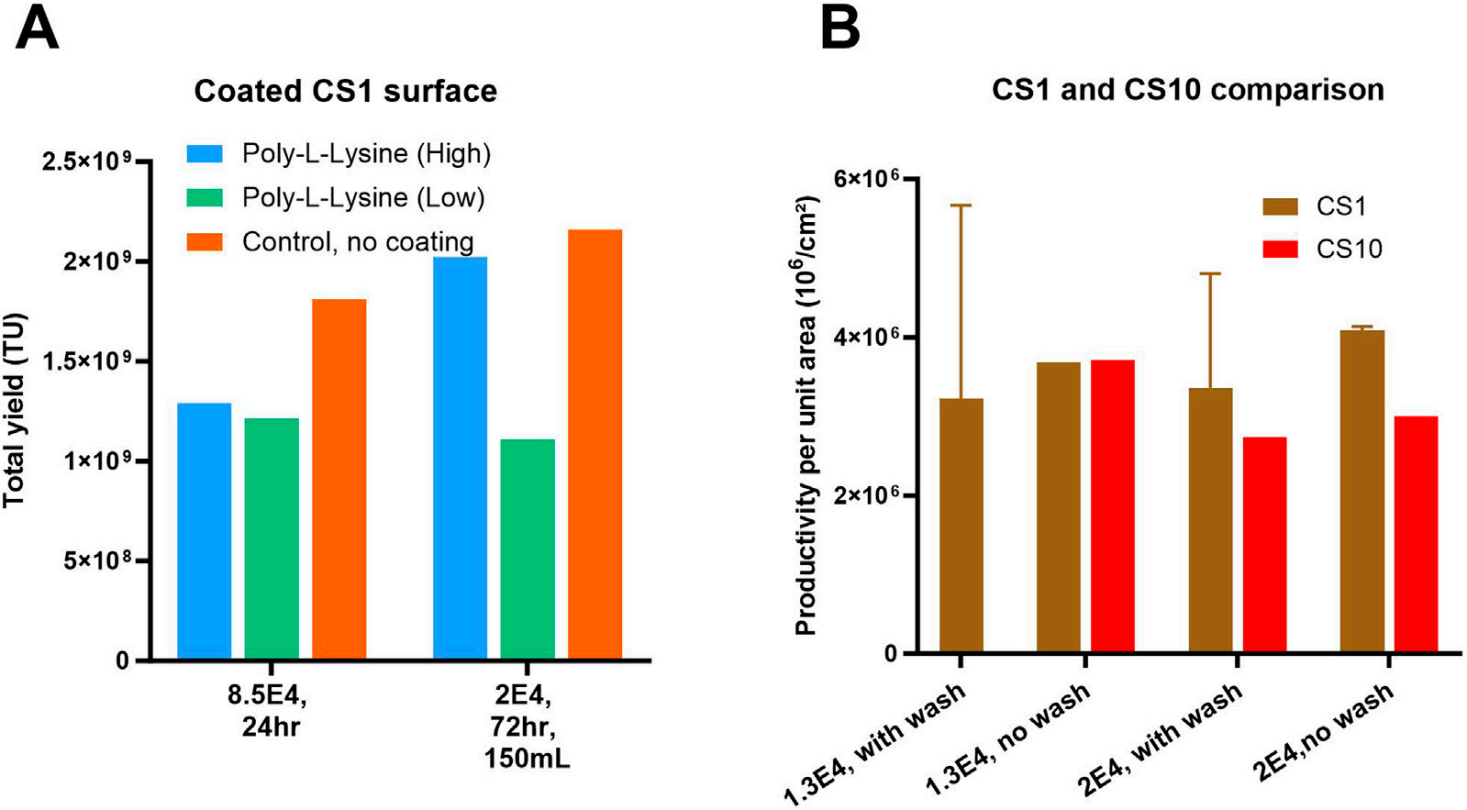
Optimization of LV production in flatware, CS1 system: **(A)** Effect of tissue culture surface coating and seeding density on total lentiviral (LV) yield. Total LV yield was evaluated under high-density (0.85E+05 cells/cm^2^ for 24 h) and low-density (2E+04 cells/cm^2^ for 72 h growth) seeding conditions using producer cell lines (PCLs). Surfaces were either uncoated or coated with low or high-molecular-weight poly-L-lysine (PLL). **(B)** Lentiviral (LV) productivity per unit area (TU/cm^2^) at small (CS1) and larger (CS10) scale. LV yield normalized to culture surface area (TU/cm^2^) was assessed under low (1.3E+4 cells/cm^2^) and high (2E+04 cells/cm^2^) seeding densities following 96 h of LV induction in CS1 and CS10 systems, with and without a cell wash step prior to induction. Shown are mean +SD (3 replicates).

For the optimization of lentiviral vector (LV) production in CellSTACKS^®^ various seeding densities, induction methods, and chemical coatings of the flatware layer were evaluated using producer cell lines of WAS construct-1. The uncoated CellSTACK (CS1), seeded at a higher density of 8.5E+04 cells/cm^2^ and induced 24 h post-seeding (hps) with a wash and 100 mL volume, served as the “control” and achieved over 50% cell layer confluency at the induction time point. To achieve similar cell layer confluency at induction, lower seeding densities of 1.3E+04 and 2E+04 cells/cm^2^ with cell growth phases of 72 or 96 h were investigated. Figure 4A demonstrates that the uncoated control yielded higher number of transducing units compared to Poly-L-lysine (high or low molecular weight) coated flatware conditions. The anticipated benefits of coating with Poly-L-lysine, such as reduced cell detachment, were not observed in the number of harvests and total TU yield. Results also indicated that the lower seeding density of 2E+04 cells/cm^2^ (with induction at 72 hps and 150 mL perfusion volume) on uncoated CS1 yield higher total TU compared to the control (8.5E+04 cells/cm^2^). To investigate the benefits of seeding density and the impact of process scale-up with the aim to reduce laborious steps, optimization by varying seeding density and influence of wash step at induction was explored in CS1 and CS10. Figure 4B shows that conditions without a wash step performed the best in both systems. All conditions with a wash step showed high variation in TU yields, potentially due to high cell detachment and cell layer disturbance. Additionally, the wide error bars on the graph indicate that CS1 was highly prone to handling issues and operator-related cell detachment during washing steps. Figure 4B shows that, on average, CS10 yields 20%–30% lower TUs per unit area compared to CS1, potentially due to slower equilibration of nutrients and gas exchange in the multiple layers of CS10.

Results in Figure 4 indicate that the lower seeding density of 1.3 E+04 and 2E+04 cells/cm^2^ and a growth phase of 96 h was optimal for LV production. Thus, the process that yielded high TUs and decently reproducible results, CS10 with 2E+04 cells/cm^2^ seeding density, 96 hps induction without a wash step, was chosen for the final pilot-scale head-to-head comparison. Overall, we have concluded that LV production processes using flatware systems have limitations in reproducibility due to operator-related process variability and are labor-intensive, especially for large-scale processes requiring the use of multiple CellSTACKS^®^.

### Comparison of iCELLis™ Nano, Scale-X™ Hydro, and CellSTACK LV production processes

Adherent bioreactors can be utilized to produce LV in a highly controlled environment compared to CellSTACKS^®^. Therefore, LV production was compared across CellSTACKS^®^ and two adherent bioreactors (iCELLis™ and Univercells Technologies™) to evaluate and compare the LV process yield, process length and robustness. The small-scale technological evaluation for a stable LV production platform comprised of six different processes of iCELLis™ Nano 4 m^2^ bioreactor (iC01, iC02, iC03, iC04, iC05 and iC06), two processes of Scale-X™ Hydro 2.4 m^2^ bioreactor (S05, S08) and one at-scale CellSTACK run with 16 units of CS10 (16CS10 with surface area 10.17 m^2^) seeded at 8.5E+04 cells/cm^2^ and induced with wash at 24 hps for LV production. All runs were seeded with the same producer cell line of WAS construct-1 for comparability.

**FIGURE 5:**
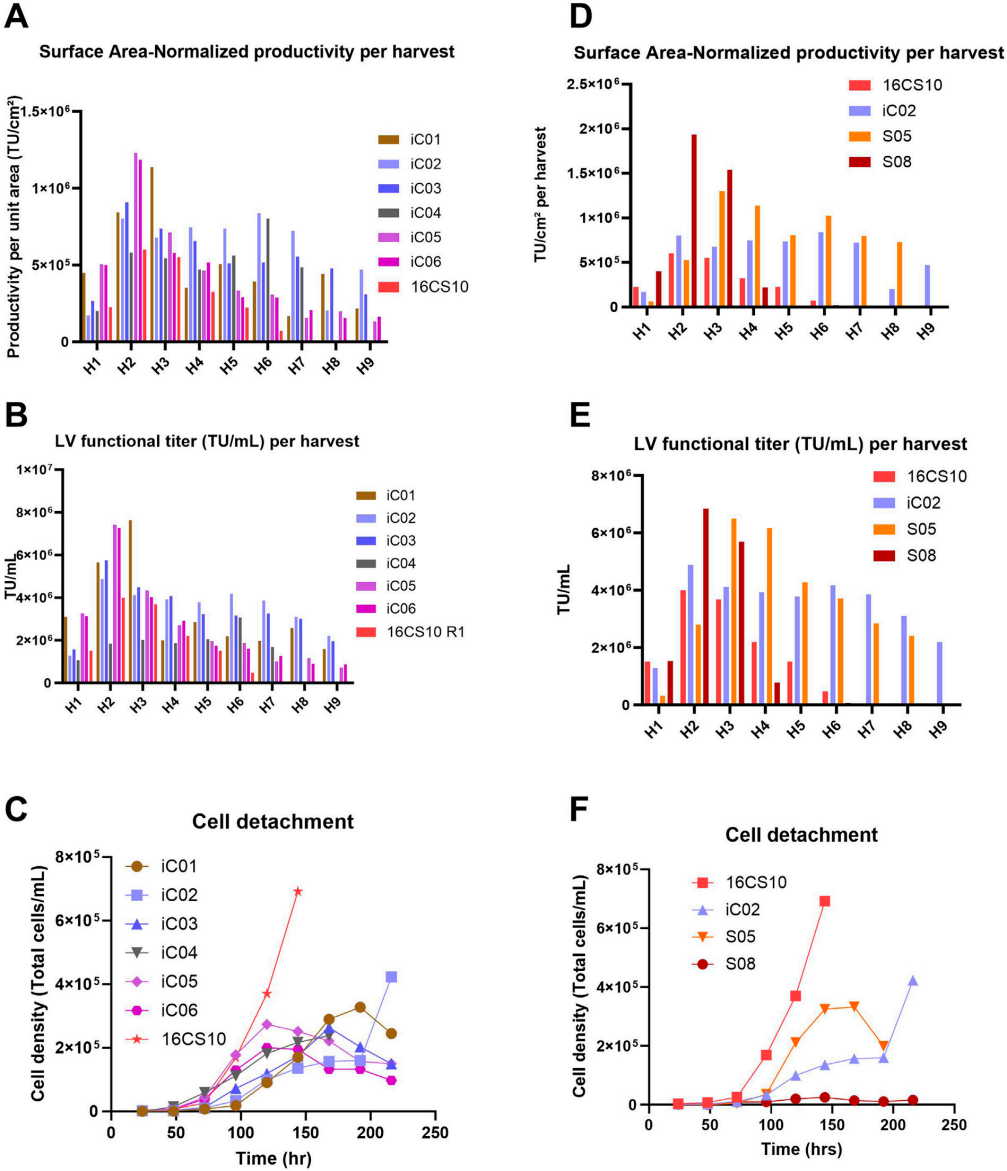
**(A–C)**: Processes run with iCELLis™ Nano 4 m^2^ fixed-bed bioreactor. The 16CS10 process consisted of 16 parallel CS10 with 6 harvests (H1 to H6), whereas all iCELLis™ runs (iC01-iC06) included 9 harvests (H1 to H9). iC01 was run without vessel wash at induction and with 0.5 M NaOH base for pH control; iC02 and iC03 were run without vessel wash and without base for pH control; iC04 was run with 1.4x higher perfusion and without vessel wash at induction; iC05 and iC06 were technical replicates where induction was performed with vessel volume wash. **(A)** Surface Area–Normalized Productivity per harvest (TU/cm^2^). **(B)** LV functional titer (TU/mL) per harvest. **(C)** Extent of cell detachment over time. Graphs **(D–F)**: Comparison of two processes using the Scale-X™ Hydro 2.4 m^2^ fixed-bed bioreactor (S05, S08) with 16x CS10 batch and the top-performing CELLis™ Nano 4 m^2^ run (iC02); S05 was directly induced without vessel wash and run in recirculation mode; S08 was induced with vessel wash and run in perfusion. **(D)** Surface Area–Normalized Productivity (TU/cm^2^) achieved per harvest. **(E)** LV functional Titer (TU/mL) achieved per harvest. **(F)** Extent of cell detachment over time.

Figures 5A–C presents a comparative analysis of various iCELLis™ (iC) processes and a CellSTACKS^®^ run (16CS10), with a graphical overview shown in Supplementary Figure S2.

**FIGURE 6:**
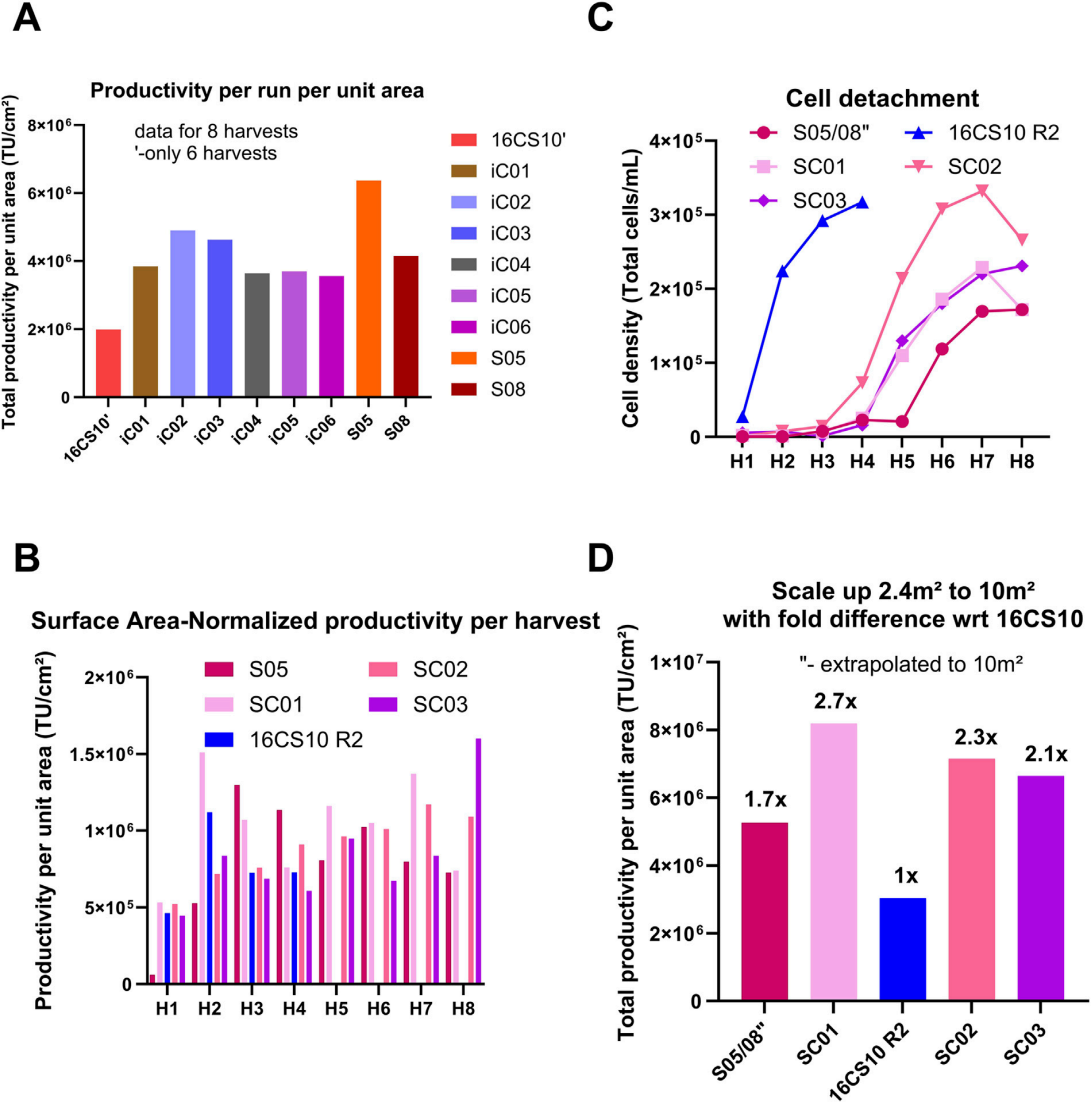
Comparison of productivity for all small-scale fixed bed bioreactor runs to each other and to 16CS10 flatware batch. **(A)** Comparison of total productivity per run per unit area across all small-scale fixed-bed bioreactor runs and the 16x CS10 flatware batch process. **(B)** Performance of Scale-X™ Carbo 10 m^2^ (pilot scale) compared with the optimized flatware process at scale (16 CS10 R2) and the average performance of S05 data represented individually from Scale-X™ Hydro 2.4 m^2^. **(C)** Extent of cell detachment over time with S05 and S08 average shown as S05/S08``. **(D)** Total TU yield normalized by surface area to compare extrapolated S05/08 performance with Scale-X™ Carbo 10 m^2^ runs. TU yields from S05/08 runs (Scale-X™ Hydro 2.4 m^2^) were extrapolated to 10 m^2^ and normalized by area, then compared to three Scale-X™ Carbo 10 m^2^ batches (SC01, SC02, SC03). SC01 was run head-to-head with the optimized 16× CS10 R2 batch, which used an equivalent surface area for LV production in the flatware system.

iC01: This process showed a three-fold decline in infectious titer (TU/mL) from the third to the fourth harvest, coinciding with the addition of a base for pH control (setpoint of 6.9 ± 0.15 during the LV production phase) starting from the second harvest day (Figures 5A,B; Supplementary Figure S3A). iC02 and iC03: These runs were operated with the same process parameters except for the pH setpoints, 7.2 for iC02 and 7.4 for iC03. Induction was performed without vessel volume exchange, and the perfusion volume was moderately increased for iC03 to prevent the pH from dropping below 6.75. Both processes showed consistent LV productivity and yields (Figures 5A,B) and exhibited low cell detachment (Figure 5C). iC04: This run was conducted in recirculation mode with a higher perfusion volume of 0.19 mL/cm^2^ from the second harvest onwards to reduce LV retention on cells and improve oxygenation. There was no increase in TU productivity per unit area (Figures 5A, 6A). The amount of LV produced per unit volume (TU/mL) was lower, suggesting dilution of the produced LV (Figure 5B). iC05 and iC06: These were technical replicates with induction involving vessel volume exchange to instantly remove residual doxycycline. Technical replicate runs showed good reproducibility and peak productivity was observed at the second and third harvests (Figures 5A,B), after which productivity drastically reduced. The peak titer at the second harvest was higher than in any other process. Cell detachment from the fixed bed was gradual (Figure 5C), with peak detachment at 2E+05 cells/mL, compared to high detachments in the 16CS10 batch of up to 7E+05 cells/mL. The TU/mL per harvest was higher for all iCELLis™ processes (except iC04) compared to CS10s.

Figures 5D–F summarizes the results from Scale-X™Hydro processes, Runs S05 and S08.

Run S08: Operated in perfusion mode with vessel volume exchange, this run showed a similar trend to iC05 and iC06, with peak titers at the second and third harvests before declining seven-fold. It exhibited the lowest cell detachment (Figure 5F) compared to other runs. Run S05: This run operated in recirculation without vessel wash at media exchange rate of 0.19 mL/cm^2^ achieved higher total TU and maintained high productivity (TU/cm^2^ per harvest, Figure 5D) over the process duration compared to all other processes. This is attributed to a higher media exchange volume of 0.19 mL/cm^2^, higher oxygen mass transfer coefficients (kLa) and steady, constant LV production in daily harvests. The glucose trend (Supplementary Figure S4C) shows that glucose levels were generally lower for S05 than for iC02, which had 0.16 mL media/cm^2^ in perfusion, while lactate trends (Supplementary Figure S4D) remained similar. This suggests that more glucose may have been metabolized in S05 towards LV production. Further pH and pCO_2_ were lower in SCO5 (Supplementary Figures S4A,F). All these led to a 20% higher total TU yield/cm^2^ (Figure 6A) in S05 compared to the best iCELLis™ Nano 4 m^2^ run (iC02) in total TU yield (seven harvests). Additionally, Figure 6A shows that the total TU yield/cm^2^ for both S05 and S08 is higher than for 16CS10. In conclusion, the S05 process performed better than 16CS10, iC01-iC06, and S08.

### Comparative analysis at pilot-scale for Scale-X™ carbo and flatware systems in LV production

A recirculation process such as S05, is complex for scale-up and has a contamination risk due to open manipulations of the media container for harvesting. Hence it was hypothesized, if the Scale-X™ Hydro bioreactor was operated in perfusion with induction performed without a vessel wash, similar or better process yields than iC02 could be expected, potentially resulting in the appropriate process design for large scale-up for stable LV production. Due to the positive outcomes with Scale-X™ Hydro, including higher total TU yield per unit area, better mixing characteristics, and the availability of a pilot-scale size, the subsequent scale-up of Scale-X™ Hydro technology led to the investigation of the Scale-X™ Carbo 10 m^2^. Three processes in the Scale-X™ Carbo 10 m^2^ system (SC01, SC02, SC03) were operated in perfusion mode, with induction performed without a wash step. The comparative analysis of these runs with flatware (16CS10R2, seeded at 1.3E+04 cells/cm^2^) and Scale-X™ Hydro (2.4 m^2^) systems is illustrated in Figures 6B–D.

SC01: The initial Scale-X™ Carbo process (run SC01) demonstrated a similar TU/cm^2^ per harvest as run S05 and a higher TU/cm^2^ compared to 16CS10R2, although it had a significantly longer process duration. The cell detachment profiles for runs SC01, SC02, and SC03 were notably lower than in the 16CS10R2 batch (Figure 6C).

Total TU Yield: Figure 6D shows the total TU yield per run normalized by area (cm^2^). Using the yield from 16CS10R2 as a reference (1-fold), the Scale-X™ Carbo 10 m^2^ SC01 achieved a 2.7-fold higher TU yield, a result consistently reproduced in SC02 and SC03. The results from runs S05 and S08 were averaged and extrapolated to a 10 m^2^ scale, yielding a theoretical 1.7-fold higher TU yield than 16CS10R2, compared to the 2.7-fold increase observed for SC01 at pilot scale. Cell Detachment and Nuclei Counts: SC02 had more cell detachment compared to other runs in Scale-X™ Carbo, resulting in a lower count of attached cells as determined by nuclei counts at the last harvest (Supplementary Figure S5B). Nevertheless, Supplementary Figure S5B shows reproducible nuclei counts from SC01, SC02, and SC03, indicating healthy cell attachment and growth on the fixed bed throughout the process duration. Oxygenation and CO2 Trends: The pO2 trend indicates adequate oxygenation in this system (Supplementary Figure S5E). The pCO2 trends were generally higher than those of the small-scale counterpart (Supplementary Figure S5F vs. Supplementary Figure S4F), likely due to the approximately four-fold higher total cell count in the vessel at pilot scale. Glucose Availability: The higher glucose availability in all three runs, approximately 2 g/L (Supplementary Figure S5C), can be attributed to the higher media perfusion rate of 0.23 mL/cm^2^, which is 20% higher than that in S05 (0.19 mL/cm^2^). In summary, the Scale-X™ Carbo 10 m^2^ system demonstrated superior performance in terms of TU yield and cell attachment compared to the flatware and Scale-X™ Hydro systems.

### Comparative evaluation of Scale-X™ carbo bioreactors for LV production using polyclonal vs. monoclonal stable producer cell

This study aimed to assess the suitability of various versions of Scale-X™ Carbo 10 m^2^ consumables as a manufacturing platform for LV production, using different stable producer cell lines generated at different times using WAS constructs 1, 2 and 3. The Scale-X™ Carbo 10 m^2^ versions (V1, V1.4, and V2) were assessed, each featuring minor differences in stirrer configuration, manifold and head plate design, and consumable readiness for use. The process parameters remained similar, to SC01, SC02, and SC03, with minor differences in volume of media perfused during induction and harvest two onwards (26L for V2 instead of 23L for V1 and V1.4) and various seeding densities employed to ensure an induction density of >1.80E+05 cells/cm^2^ is achieved with the various producer cell lines.

**FIGURE 7:**
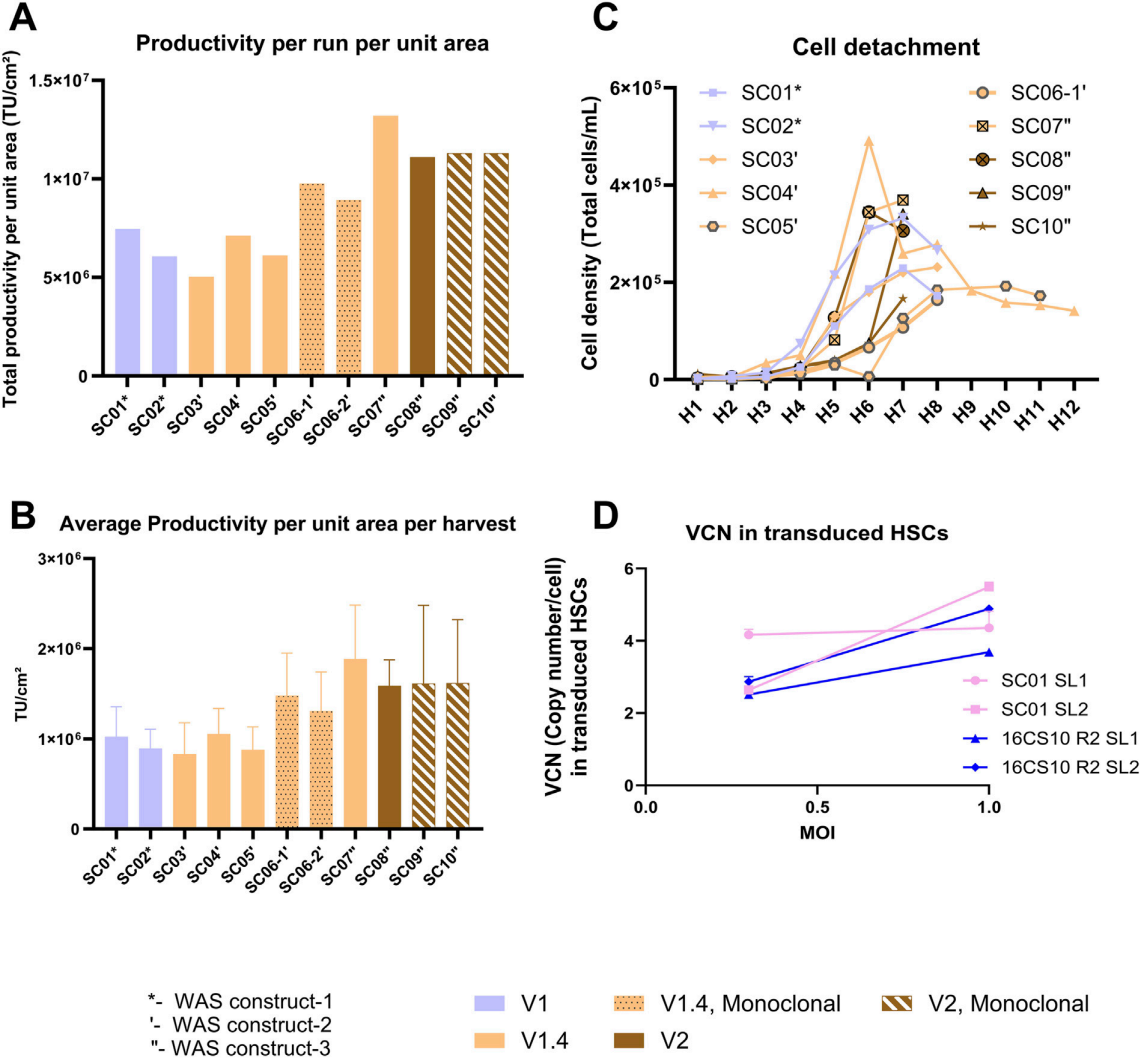
Summary outcomes from up to 10 different runs of Scale-X™ Carbo 10 m^2^ with various versions of fixed-bed consumable (V1, V1.4, and V2), and with stable producer cells from various WAS constructs generated over time (depicted by *-construct 1, ‘-construct 2, “-construct 3). The dotted bar and striped bar depict data from runs with monoclonal stable producer cell lines. **(A)** Comparison of TU yields normalized by area per run. **(B)** Average TU/cm^2^ achieved per harvest. **(C)** Extent of cell detachment over time (H1 to H8). **(D)** LV transduction capacity in CD34^+^ HSCs. LV produced from designated sublots and processes were used to transduce donor derived CD34^+^ HSCs at MOI of 0.3 and 1. VCN was determined as described in the Method section. Data are presented as means +/- SD.

Figures 7A,B summarizes the outcomes showing comparable TU yields and similar cell detachment profiles (Figure 7C), across all runs with the three WAS constructs, confirming overall process robustness across different bioreactor versions. Processes utilizing monoclonal cell lines (Runs SC06-1′, SC06-2′, SC09″, SC10″) yielded comparable or higher TUs with respect to their corresponding polyclonal cell line-based counterparts (Runs SC03′, SC04′, SC05′, SC07″, SC08″). Metabolite profile trends for pH, glucose and lactate were consistent across all runs (Supplementary Figure S6).

### Comparison of transduction capacity of LV on CD34^+^ HSCs

Additionally, the transduction capacity of LV originating from different pilot-scale production systems was evaluated using CD34^+^ HSCs. LV produced in CellSTACKS^®^ (16CS10 R2) and in Scale-X™ Carbo 10 m^2^ (SC01) show comparable CD34^+^ HSCs transduction efficiency, demonstrating a dose-response (Figure 7D). Remarkably high VCNs were achieved, ranging from 2 to 5 VCN/cell at MOI of 0.3 and 1. This outcome is highly relevant for clinical *ex-vivo* cell-based therapies. While LV produced from harvest 1-2 of SC01 achieved exceptionally high VCN of 4 at MOI of 0.3, for harvest 3-4, the VCN achieved was very similar to that of 16CS10 R2 harvest 1-2 and harvest 3-4 at MOIs of 0.3 and 1. This suggested that LV produced in Flatware has comparable LV quality to that in Scale-X™ Carbo 10 m^2^, despite higher cell detachment at harvests 3-4 and potential host cell-related impurities. The Scale-X™ Carbo 10 m^2^ process discussed in this study achieved a total of 1.13E+12 TU per run (7 harvests). With a 30% DSP yield post-purification, approximately 3.4E+11 TUs could be achieved, sufficient to treat about 140 patients with WAS at a dose of 10 MOI and 5E+06 CD34^+^ cells per kg body weight for a 50 kg individual (Tables 2, 3).

**TABLE 2:** Cell/kg dose and TU requirement per patient at low, mid and high estimate.

Considerations	Low estimate	Mid estimate	High estimate
Cells/Kg dose	2E+06	5E+06	1.50E+07
Max mass (kg)	50	50	50
MOI	10	10	10
TU Needed Per Patient	1.00E+09	2.50E+09	7.50E+09

**TABLE 3:** Comparison of potential LV doses from LV production yield per run for CD34^+^ cells transduction.

Vector batch	Cell stack	iCELLis™ Nano	Scale-X™ hydro	Scale-™ carbo
CellStack #	16	1	1	1
Surface Area (m^2^)	10	4	2.4	10
Total TU/ run	3E+11	1.57E+11	6.37E+11	1.13E+12
Concentration TU/ run (30% recovery after DSP)	9E+10	4.71E+10	1.91E+11	3.41E+11
# of Doses (Low Estimate)	90.00	47.1	191.1	351
# of Doses (Mid Estimate)	0.03	18.84	76.44	140.4
# of Doses (High Estimate)	0.08	6.28	25.48	46.8

## Discussion

The continuous increase in LV-based clinical trials for gene therapy enhance the challenge and necessity for developing reproducible, scalable and cost-efficient LV manufacturing process with superior quality (Arrasate et al., 2024; Van der Loo and Wright, 2016). Stable producer cell lines enable large-scale LV production for clinical trials, consistently achieving high LV titers and superior quality with lower cost compared to transient transfection methods (Martínez-Molina et al., 2020). Creating a stable producer cell line from stable PCLs requires high transfection efficiency, minimal cell toxicity, and high reproducibility in transfection (Kim and Eberwine, 2010). Concatemers offer advantages over traditional plasmids by enhancing transgene expression and transfected cell numbers while promoting multicopy integration of vector genome cassettes, leading to high-titer producer cell lines (Leahy et al., 1997; Maucksch et al., 2009; Throm et al., 2009). In a previous study, GFP concatemer-transfected clones generated substantially higher LV titers, reaching 1.76E7 TU/mL, compared to 1.8 E6 TU/mL in plasmid-transfected clones (Throm et al., 2009). This trend aligns with our studies, where GFP stable producer cell line generated by concatemer transfection achieved bioprocess LV titers exceeding 2E7 TU/mL at harvest 2 (unpublished data). Lipofectamine™ 3,000, a liposomal transfection reagent, showed superior efficiency in concatemer array transfection, leading to stable gene integration in our generated producer cells. High levels of EGFP were detected in HEK293 cells transfected with Lipofectamine 3,000 compared to Lipofectamine 2000 or FuGENE 6 reagents in HEK293 cells (Shi et al., 2018) which correlates to our study where the percent GFP cells, and the MFI of GFP+ cells increased with lipofectamine 3,000 compared to other transfection reagents. This can be attributed to Lipofectamine 3,000’s ability to enhance gene transfection by preventing DNA degradation in endosomes and facilitating its transport to the nucleus (FitzGerald et al., 2020).

After successfully identifying the optimal transfection condition, we evaluated the impact of different adherent PCLs (GPRG vs. GPRTG) on LV titer and LV quality. Both PCLs were developed by sequential introduction of transgenes expressing the essential components for LV production using self-inactivating (SIN) murine leukemia viruses (MLV). The GPRTG cell line also expresses the Tat protein upon induction, and the presence of Rev ensures efficient production of Tat and other viral proteins unlike the GPRG cell line which expresses only Rev and no Tat upon induction (Bonner et al., 2015; Throm et al., 2009) For these comparisons, GFP was used as a control GOI (plasmid size: 8.3kb, gene size: 720 bp) alongside WAS construct- 1 (plasmid size: 9.6kb, gene size: 1,512 bp), thereby assessing the influence of construct size on LV titer. GPRTG-based producer cells outperformed GPRG-based producer cells in LV infectious titer, RNA content, p24 expression, CD34^+^ transduction capacity along with better 5′ to 3′ ratio of RNA, for both GOI. Autologous gene therapy with LV is often hindered by low HSC transduction efficiency and limited vector supply (Jang et al., 2020; Tajer et al., 2024). LV from GPRTG-based cells achieved 3–4 times higher VCNs in CD34^+^ HSCs in comparison to LV from GPRG at similar MOIs. This correlates with a previous study that showed GPRTG produced vectors with enhanced transduction efficiency, resulting in a 2-fold increase in vector-positive colonies and a 3-5-fold increase in VCN compared to GPRG (Bonner et al., 2015). The differences in LV production efficiency and quality attributes between GPRG and GPRTG PCLs, which result in GPRTG-derived LV having superior transduction of HSCs are likely due to variations in the copy numbers and integration loci of the integrated expression cassettes for the packaging elements. GPRG contains 3 copies of gag-pol, 4 copies of tTA, 11 copies of rev, 8 copies of VSV-G, and no TAT. In contrast, GPRTG has 2 copies of gag-pol, 6 copies of tTA, 3 copies of rev, 2 copies of VSV-G, and 1 copy of TAT (Roescheise et al., 2025). Additionally, it has been shown that rev protein expression is lower in the GPRTG compared to GPRG (Bonner et al., 2015). Tat which is only expressed in GPRTG cell line enhances transcriptional elongation and gene expression by modulating key cellular pathways, increasing early mRNA levels, and enabling Rev function, likely resulting in in increased virus production in the GPRTG cell line (Clark et al., 2017; Feinberg et al., 1991). TLA sequencing of the GPRG and GPRTG packaging cell lines provided high-resolution mapping of genomic integration sites and confirmed the structural integrity of the packaging elements. The method effectively identified multiple integration sites per element and verified the presence of full-length, mutation-free sequences. These findings were further supported by ddPCR-based copy number analysis, which confirmed at least one functional copy of each packaging component per cell line. Supplementary Table 1 presents the evaluation of packaging element integration and copy number in the final top monoclonal clone using TLA and ddPCR. The combined data demonstrate stable genomic incorporation and support the consistent expression of packaging elements, reinforcing the suitability of these lines for reliable lentiviral vector production.

Despite successful transfection of stable PCL, LV titer and LV quality attributes can additionally be influenced from plasmid design, construct size and packaging efficiency based on the stably expressed packaging elements (Sweeney and Vink, 2021). In addition to cell line and process optimization, several molecular strategies have been described to enhance LV yield and stability. These include codon optimization of packaging elements, the use of strong constitutive or inducible promoters, and incorporation of post-transcriptional regulatory elements such as WPRE variants (Kotsopoulou et al., 2000; Qin et al., 2010; Chang and Sadelain, 2007). Furthermore, vector genome design such as the use of self-inactivating (SIN) LTRs and chromatin insulators can improve transcriptional activity and reduce silencing, thereby supporting sustained vector production. While capsid engineering has shown promise in other vector systems such as AAV (Meng et al., 2024; Xie et al., 2023), its applicability to LVVs remains limited due to structural and mechanistic differences.

The long-term stability of lentiviral vector (LVV) producer clones is a critical factor in ensuring consistent vector yield and quality for clinical manufacturing. As highlighted by Arrasate et al. (2024), maintaining LV stability remains a significant challenge in LV production. In alignment with this, the ICH Q5D guideline (International Council for Harmonisation, 1998) emphasizes the importance of cell substrate stability to ensure consistent production of the intended product and preservation of production capacity during storage. To mimic the establishment of a GMP-compliant Master Cell Bank (MCB) and Working Cell Bank (WCB) as well as pilot-scale lentiviral production, an extended cultivation period of at least 2 months was evaluated for our generated 4 monoclonal clones with 8–9 LV production runs per clone. Three clones maintained stable titers (≤25% reduction), while two showed ∼50% decline, highlighting variability in long-term productivity. These findings are consistent with previous reports highlighting the inherent heterogeneity among monoclonal LV producer lines. Klimpel et al. (2024) assessed six clones over 71 days of continuous passage; only one showed a notable drop in productivity after 43 days. Although numerous lentiviral vector producer cell lines have been developed over the past decade, data on their long-term stability remain scarce or entirely absent. The decline in titer observed in some clones may result from epigenetic silencing, vector rearrangements, reduced copy numbers, or suboptimal integration sites. TLA analysis, as applied in this study, offers valuable insight into integrity of integration sites and supports the identification of genomically stable clones. While full coverage of all plasmid elements was not achieved, TLA sequencing confirmed the structural integrity of the WAS transgene throughout the stability study, with no detected mutations or rearrangements. Importantly, the cultivation protocol employed in the stability study comprising continuous doxycycline induction, biweekly medium exchange, and routine subculturing closely reflects conditions used in GMP-compliant manufacturing workflows. The sustained high titers achieved by several clones under these conditions demonstrate their suitability for the generation of Master and Working Cell Banks. Furthermore, the early onset of vector production (as early as 10 days post-thaw) and consistently high cell viability (>95%) throughout the study highlight the robustness and reliability of the selected clones. Thus, this stability study confirms that with appropriate clone selection and process control, long-term LV production can be achieved with minimal loss in productivity. These findings support the feasibility of using monoclonal producer lines for clinical-grade vector manufacturing and align with industry standards established by leading gene therapy developers.

Previous studies reported that transient transfection with GFP construct in flatware process yielded 1E+06 TU/mL (Ausubel et al., 2012), and stable GFP LV production yielded above 1E+06 TU/mL (Tomás et al., 2018). Production costs for GMP-grade plasmids for transient transfection are higher, and the transient LV process exhibits batch variability due to varied transfection efficiency, leading to inconsistent yields and quality of the produced virus (Comisel et al., 2021; Perry and Rayat, 2021). Once the stable producer cell line is established, it eliminates the need for repeated purchases of plasmids and transfection reagents, reducing overall production costs and enabling consistent and robust LV production. The stability and high titers observed in our generated stable WAS producer cells can be attributed to the combined effects of concatemer formation during transfection, the use of effective transfection reagents for gene integration, and the inherent genetic disposition of the GPRTG packaging cells.

One prevalent issue in the production of lentiviral vectors (LV) using adherent HEK293T cells in flatware systems is cell detachment. This phenomenon reduces the duration of the production process and adversely affects viral yields (Noguchi et al., 2023). In our generated producer cells (GPRTG-GFP, GPRTG-WAS), we observed a correlation between infectious titer and cell detachment. High LV expression, as seen in the GPRTG cell line, led to increased cell detachment in flatware, thereby reducing the process length to 2 to 4 harvests, unlike GPRG-producer cells, which allowed up to 10 harvests. The high infectious titers of the GPRTG cells might be causing cell detachment by increasing retro-transduction of producer cells by the expressed LV through interaction of VSVG of the LV with the LDLR receptors of the producer cells (Banos-Mateos et al., 2024). Although Poly-L-lysine coating is known to enhance cell adhesion by creating a positively charged surface (Faussner et al., 2022), it was not beneficial in our study for preventing cell detachment. Apart from coating, we improved the flatware process by introducing a 96 h growth phase before seeding for LV production. This approach significantly reduced the inoculum required for pilot-scale LV production by 75%–85% (seeding 16x CS10 at 1.3E+04 to 2E+04 cells/cm^2^ required only 0.46x CS10 to 0.71x CS10, compared to 3x CS10 at 0.85E+05 cells/cm^2^), thereby reducing time, resources, and costs for medium and FBS. While Flatware systems are useful for quick early material production, daily manual manipulations can lead to operator variability, increased contamination risks, and high cell detachment compared to bioreactor processes, highlighting the need for a robust and controlled system. Continuous LV production with suspension cell lines requires complex setups, suitable cell separation technologies and tailored purification methods to generate high quality and less impure LV (Klimpel et al., 2023; Knowles et al., 2013; Leinonen et al., 2020; Olgun et al., 2019; Segura et al., 2006). Conversely, adherent cell bioreactors offer a simplified solution to run a process in perfusion. Hence, adherent bioreactors utilizing stable producer cell lines facilitate scalable, high-quality lentiviral (LV) production, making them ideal for clinical applications. We optimized small-scale adherent bioreactor processes for lentiviral vector (LV) production using the CELLis™ Nano (4 m^2^) and Scale-X™ Hydro bioreactor (2.4 m^2^) systems. The flatware LV production process with a growth phase served as the baseline for this optimization. iCELLis™ Nano bioreactors have previously been used for both transient and stable LV productions (Powers et al., 2020; Stibbs et al., 2024; Valkama et al., 2018). A transient perfusion process in the iCELLis™ system resulted in three harvests, with peak titers at 24 h and a gradual decrease at 48–72 h post-transfection, yielding total transducing units (TU) per run of up to 5.23E+04 to 3.7E+05 TU/cm^2^. A study optimizing iCELLis™ Nano (0.53 and 2.6 m^2^) in a semi-perfusion process using stable producer cell lines identified fetal bovine serum concentration, pH after induction, and timing of induction as critical parameters and optimizing these would lead to high viral yields above 2E7 TU/mL (Powers et al., 2020). The highest viral yields were observed at pH 6.6 or 6.8, although the role of pH on viral titer remains unclear. However, lentiviral vectors produced at different pH levels had no impact on LV transducibility of CD34^+^ HSC (Powers et al., 2020). Another study on continuous LV manufacturing in the iCELLis™ system showed that reducing pH from 7.20 to 6.85 along with high perfusion rate of 1.5 VVD improved total TU yield per cm^2^ from 6.19E4 per cm^2^ to 1.07E5 per cm^2^ (Stibbs et al., 2024). This transient perfusion process yielded 5.23E+04 to 3.7E+05 TU/cm^2^ over three harvests, with peak titers at 24 h. However, cells in the lower fixed-bed formed clusters, hindering access to the transfection reagent (Valkama et al., 2018).

Limited studies exist on the Scale-X™ bioreactor system, but it has shown equal or better efficiency in producing lentiviral and adenoviral vectors compared to the iCELLis™ Nano system. The Scale-X™ Hydro bioreactor yielded 9.8E+05 TU/cm^2^ LV per run versus 4.7E+05 TU/cm^2^ LV in iCELLis™, with excellent cell growth and uniform distribution. Adenoviral vector productivity was 1.11E+11 vp/mL in Scale-X™ Hydro, compared to 8.53E+10 vp/mL in iCELLis™ Nano (Leinonen et al., 2020).

Our process optimization with small-scale bioreactors demonstrated that fixed-bed bioreactor-based LV production processes achieve enhanced yields when operating at lower pH set points, specifically from 7.20 to 6.85. In this study, three LV production processes were evaluated using small-scale bioreactor systems: recirculation, continuous perfusion with vessel volume exchange at induction, and continuous perfusion without vessel volume exchange at induction. Although the recirculation process achieved comparable yields to the perfusion process, it was deemed unsuitable for scale-up and GMP batches due to the labor-intensive manual steps during harvest collection. The perfusion process without vessel volume exchange exhibited high and consistent titers, resulting in 9 harvest collections (the average total TU of IC02 for harvests 1–3, 4–6, and 7–9 were 6.6E10, 9.28E10, and 5.58E10, respectively). In contrast, the perfusion process with vessel volume exchange achieved peak titers at first three harvests, but productivity declined thereafter, leading to a shorter process of 7 harvests (the average total TU of iC05 for harvests 1–3, 4–6, and 7 were 9.7E10, 4.41E10, and 1.93E10, respectively). A direct comparison of the total TU for 7 harvests showed a 28% higher yield in the perfusion process without vessel volume exchange compared to the process with vessel volume exchange. Despite having a lower overall titer compared to the process without vessel volume exchange, the perfusion process with vessel volume exchange provides high titers at earlier harvests, making it beneficial for applications requiring a high TU yield within a shorter process duration. This also coincides with the Scale-X™ Hydro 2.4 m^2^ which showed a 3-fold (2-fold with normalized media volumes) increase in average total TU at early harvests (3.10E10 for Harvests 1–3) with SC08 (recirculation with vessel volume exchange) at a high media volume of 0.28 mL/cm^2^ compared to SC05 (perfusion without vessel volume exchange) with a standard volume of 0.16 mL/cm^2^ (1.01E10).

In our iCELLis™ process, the pH level was dependent on the stage of the process. It started at 7.0 during induction and decreased to 6.7 at later harvests. The pH drop was due to cell growth, which reached a maximum at the second harvest (when the carrier was fully covered with cells). After this point, the lower pH was maintained by a continuous perfusion process. This is consistent with other studies that mention low pH conditions support higher LV titer (Powers et al., 2020; Stibbs et al., 2024). Adding a base (IC01) to maintain pH reduced the LV titer. High perfusion rates (0.19 mL/cm^2^) were not beneficial in our studies (IC04), and the overall titer was similar to the standard perfusion volume of 0.16 mL/cm^2^. When comparing iCELLis™ Nano with Scale-X™ Hydro 2.4 m^2^ process (S05), the latter showed greater robustness with consistent cell growth and nuclei counts. It achieved higher total TUs (1.36E+11 TUs per run 24% higher titer), despite having only 0.6 times the surface area of the iCELLis™ Nano 4 m^2^.

For pilot-scale production, various versions of Scale-X™ Carbo 10 m^2^ bioreactors (V1, V1.4, V2) were used with stable producer cell lines from different WAS constructs including both polyclonal and monoclonal cell lines to ensure process robustness. The Scale-X™ Carbo 10 m^2^ process, operated in perfusion mode without vessel volume exchange at induction, demonstrated process robustness and reproducibility. Further, Scale-X™ Carbo 10 m^2^ yielded significantly higher total transducing units (TUs) compared to flatware, construct 1 achieved 2.56-fold higher total TUs (8.2E11) compared to flatware 16CS10R2 (3E11). Construct 2 saw a 2.93-fold increase and construct 3 had about a 3.91-fold increase. Additionally, LV produced in Scale-X™ Carbo 10 m^2^ shows good transducibility (average bulk VCN of 4.93 at MOI 1) with excellent dose response, slightly surpassing the outcomes from CellStack preparations (average bulk VCN of 4.1 VCN at MOI 1). Achieving higher VCN at lower MOI reduces LV overload on patient cells, as higher MOI is known to cause insertional mutagenesis and cytotoxicity (Park et al., 2015). In our process, a Scale-X™ Carbo 10 m^2^ run (WAS construct-3) yielded 1.13E+12 TU per run, and with ∼30% recovery (∼3.4E+11TU) is sufficient to treat up to 140 WAS patients (50 kg each) at an MOI of 10, with 5E+06 CD34+ cells/kg (Tables 2, 3). A self-inactivating lentiviral vector using an insulated MND promoter highly analogous to our WAS constructs effectively restored WASp expression in all hematopoietic lineages in WAS knockout mice, correcting immune defects with minimal viral integration and no signs of insertional mutagenesis. In non-human primates, the vector showed stable, long-term expression, supporting its safety and efficacy for future Wiskott-Aldrich syndrome gene therapy (Singh et al., 2017). Complementing these findings, a dedicated nonclinical toxicology study was conducted to assess the transduction efficiency and hematopoietic reconstitution of the WAS lentiviral vector specifically using WAS construct-3 in a murine WAS model. The results confirmed the safety and efficacy of the approach, demonstrating robust engraftment, consistent restoration of WASp expression across multiple hematopoietic lineages, and functional immune recovery, with no evidence of toxicity, clonal dominance, or malignancies. *In vivo* results, including integration site analysis, confirm the structural and functional of the packaging elements enabled by stable genomic integration and sustained expression (unpublished).

While this study focused on adherent stable producer cell lines and fixed-bed bioreactor systems, suspension-based LV production platforms represent an important complementary approach for large-scale manufacturing. Suspension cultures offer advantages in scalability, automation, and compatibility with serum-free media. Our group has previously reported the development of stable suspension-adapted producer cell lines capable of high-titer LV production in perfusion mode (Klimpel et al., 2023; Klimpel et al., 2024; Klimpel et al., 2025). These studies also addressed key challenges such as retro-transduction of producer cells and process intensification strategies.

In conclusion, our study presented optimized methods for generating stable polyclonal and monoclonal producer cell lines that produce high LV titers from PCLs. We evaluated two PCLs (GPRG and GPRTG) in detail for the first time, using control GFP and WAS clinical constructs, in terms of titer (TU), p24, RNA content, and transduction studies. Based on infectious titer and transduction efficiency and dose-response in CD34^+^ HSCs, GPRTG was chosen for further cell line and process development activities. For the first time, the study compared traditional flatware systems with two adherent bioreactors (iCELLis™ and Univercells Technologies™) using WAS constructs as the GOI, the study developed three lentiviral (LV) processes through bioreactor optimizations: Recirculation, continuous perfusion with vessel volume exchange at induction, and continuous perfusion without vessel volume exchange at induction. Process scale-up was evaluated with Scale-X™ Carbo 10 m^2^ bioreactor, which was found to be a suitable and economical pilot-scale production technology for producing high LV in a controlled, closed system with a lower footprint with fewer batch failures and consistent quality, thereby supporting the high demand for clinical applications. The optimized LV process at a 10 m^2^ scale (Scale-X™ Carbo) was directly transferred to GMP LV production and LV production has been successfully completed. Overall, this paper proposes methods and process optimizations for generating high-titer LV, highlighting scalable technologies for producing clinical-grade LV with high transduction efficiency.

## Data Availability

All data generated or analyzed in this study are included in the published article/Supplementary Material, further inquiries can be directed to the corresponding author.

## Supplementary material

SUPPLEMENTARY FIGURE S1 Plasmid constructs **(A)** WAS construct-1, plasmid size of 9.6 kb, includes a 650 bp segment of the chicken hypersensitivity site 4 (cHS4) β-globin chromatin insulator that is inserted in the 3’LTR in reverse orientation to the viral transcript, serving as an additional safety and anti-silencing element. **(B)** WAS construct-2, with a plasmid size of 9.6 kb, contains a mutated version of the 650 bp segment of the cHS4 insulator. **(C)** WAS construct-3, with a plasmid size of 9.3 kb, includes a 400 bp segment of the cHS4 insulator. **(D)** The antibiotic-resistance plasmid (pPGK_ble), with a plasmid size of 3.8 kb, contains a Zeocin selection marker driven by the PGK promoter.

SUPPLEMENTARY FIGURE S2 LV production process flowchart illustrating three possible process designs, from seed train through harvest, for optimized processes using CellStack and adherent bioreactor systems.

SUPPLEMENTARY FIGURE S3 Daily bioreactor monitoring and metabolite profiles for various runs comparing: **(A)** pH. **(B)** Nuclei counts from carriers. **(C)** Glucose. **(D)** Lactate. **(E)** pO2. **(F)** pCO2. Trends over the process duration for all iCELLis™ Nano 4m^2^ runs (G1 - Growth day 1, G2 - Growth day 2, G3 - Growth day 3, G4 - Growth day 4, Dis - Discard, H1 to H8 - Harvest 1 to Harvest 8).

SUPPLEMENTARY FIGURE S4 Daily bioreactor monitoring and metabolite profiles for various runs comparing: **(A)** pH. **(B)** Nuclei counts from carriers. **(C)** Glucose. **(D)** Lactate. **(E)** pO2. **(F)** pCO2. Trends over the process duration for Scale-X™ Hydro 2.4m^2^ (S05 & S08) and one iCELLis™ Nano 4m^2^ (iC02) runs (G1 - Growth day 1, G2 - Growth day 2, G3 - Growth day 3, G4 - Growth day 4, Dis - Discard, H1 to H8 - Harvest 1 to Harvest 8).

SUPPLEMENTARY FIGURE S5 Daily monitoring and metabolite profiles for various runs comparing: **(A)** pH. **(B)** Nuclei counts from carriers. **(C)** Glucose. **(D)** Lactate. **(E)** pO2. **(F)** pCO2. Trends over the process duration for Scale-X™ Hydro 2.4m^2^ (average of runs S05/S08) and three Scale-X™ Carbo 10m^2^ pilot scale (SC01, SC02, SC03) as well as one pilot scale flatware run 16CS10 R2 (G1 - Growth day 1, G2 - Growth day 2, G3 - Growth day 3, G4 - Growth day 4, Dis - Discard, H1 to H8 - Harvest 1 to Harvest 8).

SUPPLEMENTARY FIGURE S6 Daily bioreactor monitoring and metabolite profiles for various runs comparing: **(A)** pH. **(B)** Nuclei counts from carriers. **(C)** Glucose. **(D)** Lactate. Trends over the process duration for ten Scale-X™ Carbo 10m^2^ pilot scale runs from various WAS constructs generated over time (depicted by *-construct 1, ‘-construct 2, “-construct 3) (SC01*, SC02*, SC03’, SC04’, SC05’, SC06’-1, SC07”, SC08”, SC09”, SC10”) (G1 - Growth day 1, G2 - Growth day 2, G3 - Growth day 3, G4 - Growth day 4, Dis - Discard, H1 to H8 - Harvest 1 to Harvest 8).

## Glossary

LV: Lentivirus
LVV: Lentiviral vector
WAS: Wiskott-Aldrich Syndrome
PCL: Packaging Cell Line
GOI: Gene of Interest
VCN: Vector Copy Number
MOI: Multiplicity of Infection
TU: Transducing Units
vp: Viral Particles
ddPCR: Digital Droplet Polymerase Chain Reaction
HEK293T: Human Embryonic Kidney 293T Cells
HSCs: Hematopoietic Stem Cells
SIN: Self-Inactivating
CMV: Cytomegalovirus
LTR: Long Terminal Repeat
PBS: Primer Binding Site
WASp: WAS protein
WHV: Woodchuck Hepatitis Virus
WPRE: Woodchuck Post-transcriptional Regulatory Element
WHX: Woodchuck Hepatitis Virus X Protein
MCB: Master Cell Bank
WCB: Working Cell Bank
TLA: Targeted Locus Amplification
NGS: Next-Generation Sequencing
MFI: Median Fluorescence Intensity
CS1/CS5/ CS10: CellSTACK 1-layer/5-layer/ 10-layer
PLL: Poly-L-lysine
DSP: Downstream Processing
**TU/cm** ^**2**^: Transducing Units per square centimeter
TU/mL: Transducing Units per milliliter
RNA: Ribonucleic Acid
DNA: Deoxyribonucleic Acid
GFP: Green Fluorescent Protein
EGFP: Enhanced Green Fluorescent Protein
FBS: Fetal Bovine Serum
DO: Dissolved Oxygen
**pCO**_**2**_: Partial Pressure of Carbon Dioxide
**pO**_**2**_: Partial Pressure of Oxygen
kLa: Oxygen Mass Transfer Coefficient
TFF: Tangential Flow Filtration
GMP: Good Manufacturing Practice
RCB: Research Cell Bank
TE: Transduction Enhancer
TU/VP: Transducing Units per Viral Particle
TU/RNA: Transducing Units per RNA copy
TU/p24: Transducing Units per p24 concentration
TU/µg: Transducing Units per microgram
