# Supplementary figures and images for: Enhancing lentiviral production for WAS gene therapy: a comparative analysis of stable producer cell lines evaluating flatware system and adherent bioreactors in perfusion mode
Source: Front Bioeng Biotechnol. 2025 Sep 5;13:1648028. doi: 10.3389/fbioe.2025.1648028 (PMC12446366; doi:10.3389/fbioe.2025.1648028)

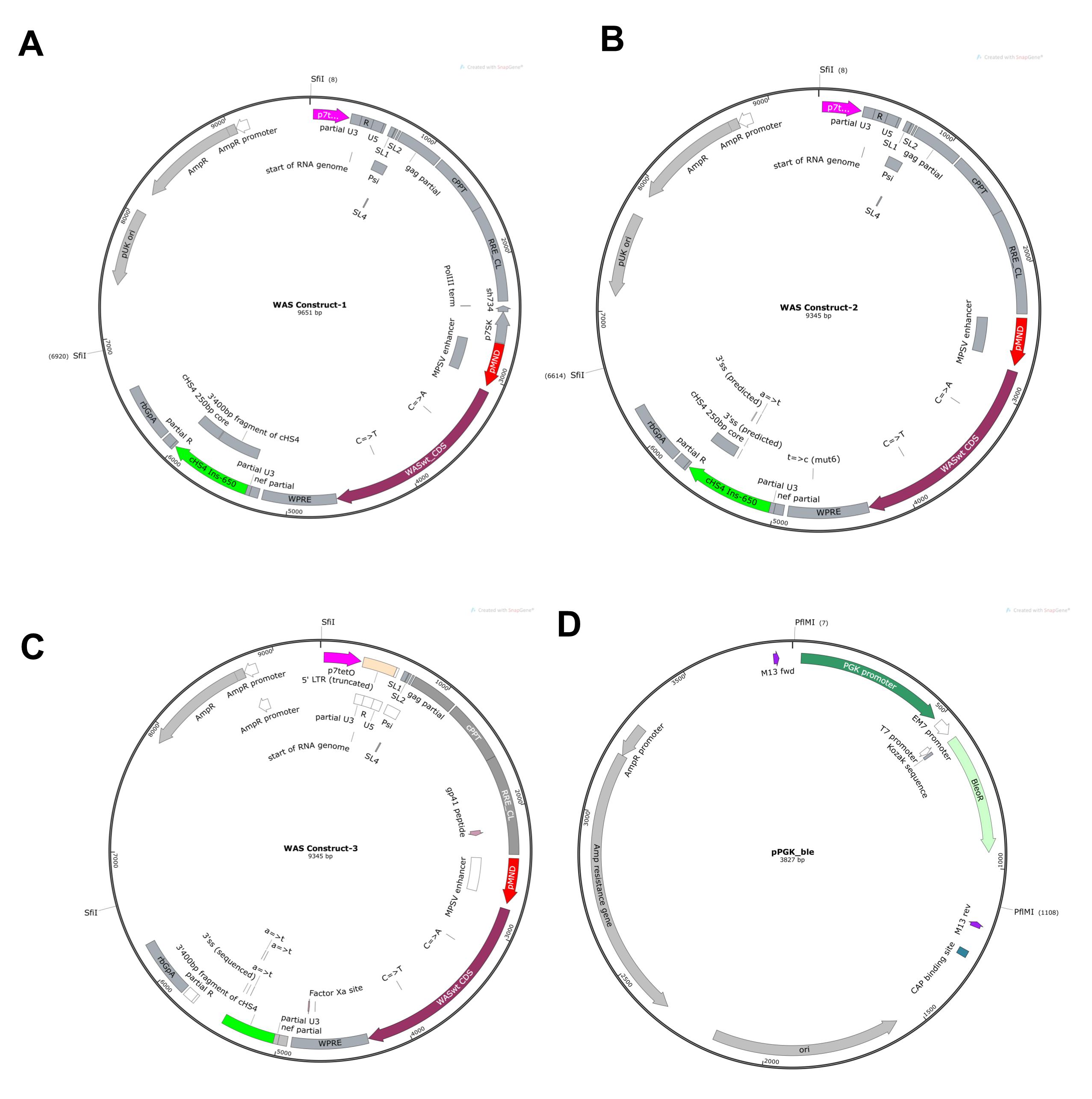

Supplement: Supplementary file 4 [file Image1.jpeg]

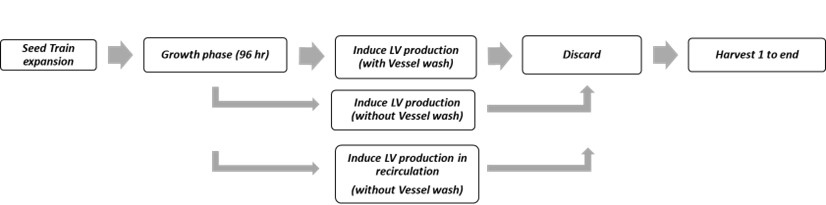

Supplement: Supplementary file 5 [file Image2.jpeg]
