# Supplementary material for: Enhancing lentiviral production for WAS gene therapy: a comparative analysis of stable producer cell lines evaluating flatware system and adherent bioreactors in perfusion mode
Source: Front Bioeng Biotechnol. 2025 Sep 5;13:1648028. doi: 10.3389/fbioe.2025.1648028 (PMC12446366; doi:10.3389/fbioe.2025.1648028)
Supplement: Supplementary file 7 [file DataSheet1.docx]

**Supplementary figure title**

Figure S1: Plasmid constructs

Figure S2: LV production process flowchart

Figure S3: Trends in pH, nuclei-counts, metabolites, and gas profile from iCELLis™ Nano (4 m²) runs

Figure S4: Trends in pH, nuclei-counts, metabolites, and gas profile from Scale-X™ Hydro (2.4 m²) runs compared to one iCELLis™ Nano (iC02) run

Figure S5: Trends in pH, nuclei-counts, metabolites, and gas profile for Scale-X™ Hydro (2.4 m²) runs compared to pilot-scale CellSTACK and Univercells Carbo (10 m²) runs

Figure S6: Trends in pH, nuclei-counts and metabolites from Univercells Carbo (10 m²) runs from various WAS constructs and polyclonal/monoclonal cell lines

**SUPPLEMENTARY FIGURES LEGEND**

**Figure S1: Plasmid constructs**

(A) WAS construct-1, plasmid size of 9.6 kb, includes a 650 bp segment of the chicken hypersensitivity site 4 (cHS4) β-globin chromatin insulator that is inserted in the 3’LTR in reverse orientation to the viral transcript, serving as an additional safety and anti-silencing element.

(B) WAS construct-2, with a plasmid size of 9.6 kb, contains a mutated version of the 650 bp segment of the cHS4 insulator.

(C) WAS construct-3, with a plasmid size of 9.3 kb, includes a 400 bp segment of the cHS4 insulator.

(D) The antibiotic-resistance plasmid (pPGK_ble), with a plasmid size of 3.8 kb, contains a Zeocin selection marker driven by the PGK promoter.

**Figure S2: LV production process flowchart**

LV production process flowchart illustrating three possible process designs, from seed train through harvest, for optimized processes using CellStack and adherent bioreactor systems

**Figure S3: Trends in pH, nuclei-counts, metabolites, and gas profile from iCELLis™ Nano (4 m²) runs**

Daily bioreactor monitoring and metabolite profiles for various runs comparing:

**(A)** pH

**(B)** Nuclei counts from carriers

**(C)** Glucose

**(D)** Lactate

**(E)** pO2

**(F)** pCO2

Trends over the process duration for all iCELLis™ Nano 4m² runs (G1 - Growth day 1, G2 - Growth day 2, G3 - Growth day 3, G4 - Growth day 4, Dis - Discard, H1 to H8 - Harvest 1 to Harvest 8).

**Figure S4: Trends in pH, nuclei-counts, metabolites, and gas profile from Scale-X™ Hydro (2.4 m²) runs compared to one iCELLis™ Nano (iC02) run**

Daily bioreactor monitoring and metabolite profiles for various runs comparing:

**(A)** pH

**(B)** Nuclei counts from carriers

**(C)** Glucose

**(D)** Lactate

**(E)** pO2

**(F)** pCO2

Trends over the process duration for Scale-X™ Hydro 2.4m² (S05 & S08) and one iCELLis™ Nano 4m² (iC02) runs (G1 - Growth day 1, G2 - Growth day 2, G3 - Growth day 3, G4 - Growth day 4, Dis - Discard, H1 to H8 - Harvest 1 to Harvest 8).

**Figure S5: Trends in pH, nuclei-counts, metabolites, and gas profile for Scale-X™ Hydro (2.4 m²) runs compared to pilot-scale CellSTACK and Univercells Carbo (10 m²) runs**

Daily monitoring and metabolite profiles for various runs comparing:

**(A)** pH

**(B)** Nuclei counts from carriers

**(C)** Glucose

**(D)** Lactate

**(E)** pO2

**(F)** pCO2

Trends over the process duration for Scale-X™ Hydro 2.4m² (average of runs S05/S08) and three Scale-X™ Carbo 10m² pilot scale (SC01, SC02, SC03) as well as one pilot scale flatware run 16CS10 R2 (G1 - Growth day 1, G2 - Growth day 2, G3 - Growth day 3, G4 - Growth day 4, Dis - Discard, H1 to H8 - Harvest 1 to Harvest 8).

**Figure S6: Trends in pH, nuclei-counts and metabolites from Univercells Carbo (10 m²) runs from various WAS constructs and polyclonal/monoclonal cell lines**

Daily bioreactor monitoring and metabolite profiles for various runs comparing:

**(A)** pH

**(B)** Nuclei counts from carriers

**(C)** Glucose

**(D)** Lactate

Trends over the process duration for ten Scale-X™ Carbo 10m² pilot scale runs from various WAS constructs generated over time (depicted by *-construct 1, ‘-construct 2, “-construct 3) (SC01*, SC02*, SC03’, SC04’, SC05’, SC06’-1, SC07”, SC08”, SC09”, SC10”) (G1 - Growth day 1, G2 - Growth day 2, G3 - Growth day 3, G4 - Growth day 4, Dis - Discard, H1 to H8 - Harvest 1 to Harvest 8).

**SUPPLEMENTARY Table 1**

**Supplementary Table 1 Evaluation of Packaging Element Integration and Copy Number in the Final Top Monoclonal Clone (GPRTG with WAS construct-3) Using TLA and ddPCR**

| Construct | TLA | ddPCR |
| --- | --- | --- |
| SFG-IC-HIVgp-Ppac2 | 2 | 1.98 GAG, 2.04 Pol |
| SFG-tc-revco | 2 | 1.55 |
| SFG-tc-tatco | 1 | 0.94 |
| SFG-tc-VSVG | 2 | 2.17 |
| SFG-tTA | 6 | 7.34 |
